# A Combination of *Hermetia illucens* Reared on Fish Waste and Poultry By-Product Meal Improves Sensory and Physicochemical Quality of Farmed Barramundi Filets

**DOI:** 10.3389/fnut.2021.788064

**Published:** 2022-01-12

**Authors:** Md Reaz Chaklader, Wing H. Chung, Janet Howieson, Ravi Fotedar

**Affiliations:** School of Molecular and Life Sciences, Curtin University, Bentley, WA, Australia

**Keywords:** *Lates calcarifer*, black soldier fly larvae, poultry by-products, circular bioeconomy, sensory evaluation, filet brightness, lipid oxidation

## Abstract

The proximate composition, sensory attributes, and shelf life of filets from barramundi, *Lates calcarifer*, were fed a fishmeal (FM) based diet (0PBM-0HI) and three test diets replacing FM protein entirely with 85% poultry by-products meal (PBM) and 15% *Hermetia illucens* (HI) larvae meal protein (85PBM-15HI), 80% PBM and 20% HI (80PBM-20HI) and 75% PBM and 25% HI (75PBM-25HI) were investigated. After a 56-day feeding trial, the crude protein, moisture, and ash percentage were unchanged while the crude lipid increased in barramundi filet when fed with PBM-HI-based diets. The increase in C12:0 (lauric acid) and C14:0 (myristic acid) resulted in an increase in the total saturated fatty acid while the monounsaturated fatty acid elevated due to an increase in C16:1n7 and C18:1cis + trans in the filet of the barramundi fed with a PBM-HI based diet. While the decrease in the total polyunsaturated fatty acid (PUFA) content in PBM-HI based fed barramundi filet was mainly due to a decrease in essential fatty acids including C20:5n3 [eicosapentaenoic acid (EPA)] and C22:6n3 [docosahexaenoic acid (DHA)] when compared with the 0PBM-0HI fed barramundi filet. The sensory quality was improved by PBM-HI-based diets, manifested by the highest scores given by the panelists. Texture profiles were not affected by diet but cohesiveness, gumminess, and chewiness decreased with increasing storage time. On days 1 and 8, skin brightness decreased in the skin of the barramundi fed with 85PBM-15HI and 80PBM-20HI compared with the skin of the 0PBM-0HI fed barramundi. Skin redness improved in fish-fed PBM-HI-based diets. The flesh brightness and yellowness increased significantly in barramundi when fed with PBM-HI-based diets. On days 1 and 4, the flesh brightness of the barramundi fed with PBM-HI-based diets demonstrated an increase compared with 0PBM-0HI. PBM-HI diets suppress lipid oxidation while lipid oxidation increased over the storage time. In summary, the improvement in sensory quality and color coupled with the suppression of rancidity in barramundi filets underpinned the potentiality of using the mixture of PBM and HI transformed from food waste in the barramundi diet to improve the filet quality and thus support sustainability and circular economy in aquaculture.

## Graphical Abstract

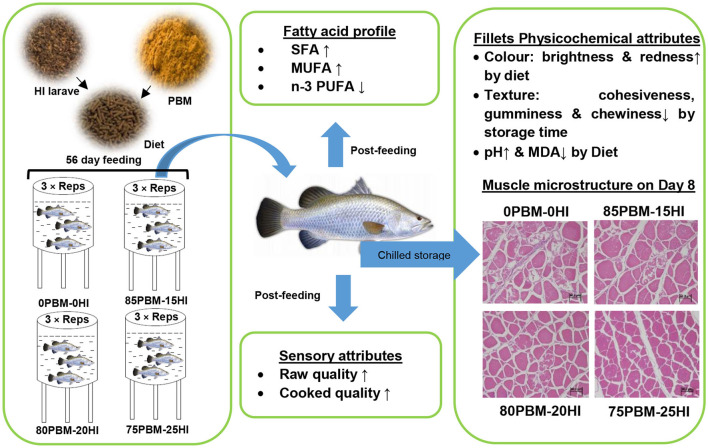

## Introduction

The rapid expansion of the global aquaculture industry has exceeded the annual growth rate of other animal protein-producing industries such as the poultry, pork, dairy, and beef industry (1). However, due to high cost and sustainability issues associated with fishmeal (FM), this resource is progressively being replaced by various plant and animal-based sources to fill the macronutrient void in aquafeed production, an approach also supporting the provision of aquaculture sustainability. The high inclusion of plant-based sources featuring low protein content, imbalanced amino acid profile, and biologically active antinutritional factors have been reported to stimulate a negative effect on carnivorous fish, such as barramundi, *Lates calcarifer* as demonstrated by a number of studies in our laboratory (2–9).

Barramundi is an important tropical food fish farmed in South East Asian countries and Australia and its 40% annual production come from captive aquaculture production among the total global harvest estimated at around 164,000 t per annum where in Australia, 60% of barramundi is produced by aquaculture out of the total national production (10–12). However, barramundi farming in Australia is heavily dependent on imported conventional protein sources, consequently reducing the profitability of barramundi (13). Recent innovations have successfully validated FM-free diets for barramundi (14, 15), however, farmed barramundi of all sizes develop blue-grayish discoloration on the dorsal area of filet flesh associated with the acceptance of consumers (16). The same authors attempted to resolve the blue-grayish problems in barramundi by dietary modifications in a trial but the problems were unresolved. The mixture of poultry by-product and black soldier fly, *Hermetia illucens* (HI) larvae meal on flesh color and other filet quality traits beyond the growth and health performance at the exclusion of FM has not been thoroughly investigated.

Poultry by-products meal (PBM) has been considered as one of the animal protein ingredients to replace FM in the diets of carnivorous and omnivorous aquaculture species (17). This is due to high production volume together with cheaper price, high protein, complete amino acid profile, a good amount of essential fatty acids, vitamins, minerals, and acceptable palatability (18–20). Nevertheless, the nutritional composition of PBM, depending on processing techniques and suppliers, can vary and often lacks essential fatty acids and amino acids. PBM has been evaluated on numerous finfish and shellfish species with various successes (20). In barramundi, PBM can completely replace FM without compromising the growth, serum metabolites, immune response, and integrity of various tissues when complemented with fish protein hydrolysate and/or insects larvae (21–24). However, the potentiality of an ingredient cannot be solely evaluated by the effects on growth and other physiological and immunological parameters as dietary alteration may stimulate alteration in organoleptic characteristics and other biochemical composition functions of fish filets. The exclusive or complete inclusion of animal protein ingredients such as PBM or meat meal had no effect on the barramundi filet texture and color (24, 25) though female tenches, *Tinca tinca* filet quality was impacted by PBM (26). Except for filet organoleptic characteristics, PBM alone reduced the retention of nutrient composition such as essential amino acid and fatty acid for barramundi (27, 28), however, such effects were not observed for other species. Nonetheless, the effects that the mixture of HI larvae meal and PBM might have on barramundi filet organoleptic traits have not yet been evaluated.

Recently, insects have received much attention in the light of circular bioeconomy principles being applied to aquaculture (29). Besides valorizing low-quality by-products or organic waste leaving less environmental footprint (30–32), HI, one of the promising insects can accumulate a good amount of proteins (31–59%) featuring minor deficiency in essential amino acids suitable for omnivorous and carnivorous fish. It is also a good source of fat (11–49%), vitamins, minerals, and even biologically active compounds such as chitin, antimicrobial peptide, and short- and medium-chain fatty acids (SCFAs and MCFAs) (33), making this insect as novel alternative protein ingredients. These biologically active compounds have been associated with improved fish health by enhancing the innate immune response and modulating the gut microbiota (34–37). However, due to lower protein levels, the utilization of full-fat HI (FHI) larvae meal is a less popular approach than the use of defatted HI larvae meal (38). It is worthy to mention that a number of recent studies have proven the ameliorative effect of full-fat HI larvae in PBM and plant protein on the health of barramundi (39) and rainbow trout (40). Also, the replacement of FM with full-fat HI larvae meal alone improved the feed utilization, gut health, and immune responses of different fish species. What is more, the utilization of full-fat HI larvae meal will reduce the additional cost incurred by the defatting process which may degrade the nutritional quality and functional compounds (38). However, evaluating filet quality traits beyond the health aspects of the fish is a research area of high interest while introducing new alternative protein ingredients in aqua diets. It has been reported that the filet qualitative traits of fish were influenced by the HI larvae, nevertheless, the effect of full-fat HI larvae meal in PBM replacing FM completely on the filet quality traits has not been investigated till now.

Besides the influence of dietary modification on filet quality, the post-harvest condition has been reported to influence the filet quality attributes (12). Hence, the enrichment of fish filet muscle with functional molecules has been suggested as a good strategy to extend the shelf-life by reducing lipid oxidation (41, 42). This was underpinned by Jones and Carton (12) who found that feeding barramundi with alpha-tocopherol acetate enriched diet retarded the lipid oxidation thereby extending shelf-life during chilled storage. Hence, complementation of PBM with full-fat HI rich in functional molecules could be an option to prevent rancidity production and associated deteriorations in farmed barramundi filets during storage conditions.

The current study integrated the findings on growth performance and organo-somatic indices, serum metabolites, gastrointestinal mucosal morphology, and gut microbiota, and immunity of the barramundi fed with the mixture of full-fat HI larvae and PBM (43), by exploring the influence of this mixture of two animal protein sources on the filet quality traits in terms of fatty acids, texture, color, and muscle structure during shelf life for 8 days.

## Materials and Methods

### Ethical Statement

The feeding trial and all the protocols conducted at the Curtin Aquatic Research Laboratory (CARL) were performed in strict compliance with the guidelines and regulations of Australia and the acts were reviewed and approved by the Curtin University Animal Ethics Committee (ARE2018-37).

### Diets, Fish Husbandry, and Sampling

The details of the diet formulations and fish rearing are reported in our earlier study (43). Briefly, four isonitrogenous and isolipidic were formulated: an FM-based diet control (0PBM-0HI) diet and three diets with the complete replacement of FM protein with 85% PBM and 15% HI larvae meal (85PBM-15HI), 80% PBM and 20% HI larvae (80PBM-20HI), and 75% PBM and 25% HI larvae (75PBM-25HI). The ingredients and chemical composition of the diets are presented in Table 1.

**Table 1:** Feed formulation and nutritional composition of control and test diets containing different proportions of poultry by-products meal (PBM) and *Hermetia illucens* (HI) larvae meal.

	**Experimental diets**				^*^Ingredients	
**^a^Ingredients (g/100 g)**	**0PBM-0HI**	**85PBM-15HI**	**80PBM-20HI**	**75PBM-25HI**	**PBM**	**FHI**
FM	72.00	0.00	0.00	0.00	-	-
PBM	0.00	60.50	56.00	53.00	-	-
Canola oil	1.00	3.00	2.00	1.10	-	-
Full-fat HI	0.00	16.70	23.00	28.70	-	-
Corn/wheat starch	7.00	8.00	5.90	6.00	-	-
Lecithin–Soy (70%)	1.00	2.00	2.00	2.00	-	-
Vitamin C	0.05	0.05	0.05	0.05	-	-
Dicalcium Phosphate	0.05	0.05	0.05	0.05	-	-
Wheat (10 CP)	16.90	6.20	7.50	6.40	-	-
Vitamin and mineral premix	0.50	0.50	0.50	0.50	-	-
Salt (NaCl)	1.00	1.00	1.00	1.00	-	-
Cod liver oil	0.50	2.00	2.00	1.20	-	-
**Nutritional composition (%)**						
Crude protein	47.88	47.76	47.36	47.41	-	-
Crude Lipid	12.59	13.29	13.78	13.41	-	-
Moisture	4.56	4.68	4.53	4.36	-	-
Ash	10.97	11.10	11.25	11.06	-	-
**Fatty acid (% of total FA)**						
C10:0	0.04	0.32	0.44	0.57	0.04	2.31
C12:0	1.01	7.51	10.42	13.70	0.09	43.05
C14:0	2.32	2.11	2.61	3.04	0.68	6.80
C16:0	20.12	16.55	16.72	16.94	21.65	11.11
C16:1n7	2.80	3.45	3.61	3.70	5.01	1.55
C17:0	1.16	0.31	0.34	0.37	0.45	0.53
C18:0	6.75	5.11	5.05	5.08	7.07	3.51
C18:1cis + trans	20.50	37.23	34.69	31.93	40.88	16.05
C18:2 cis	10.17	16.42	15.33	14.92	16.09	12.23
C18:3n3	2.22	3.91	3.61	3.34	2.41	1.65
C20:1	1.41	1.57	1.56	1.21	0.56	0.09
C20:4n6	1.95	0.77	0.77	0.77	1.67	0.05
C20:5n3	3.66	1.07	1.20	1.12	0.16	0.05
C22:4n6	1.80	0.08	0.07	0.07	0.04	0.00
C22:5n3	1.13	0.34	0.35	0.28	0.34	0.01
C22:6n3	19.39	1.35	1.25	0.88	0.25	0.01
SFA^†^	33.12	32.55	36.20	40.34	31	67.86
MUFA^‡^	25.47	42.65	40.26	37.17	46.88	17.92
PUFA[Table-fn TN4]	41.58	24.97	23.69	22.60	22.18	14.23
n-3 PUFA[Table-fn TN5]	26.95	7.04	6.86	6.16	3.33	1.75
n-6 PUFA[Table-fn TN6]	4.05	1.09	1.08	1.15	2.45	0.15

*0PBM-0HI, fishmeal based diet; 85PBM-15HI, 85% protein from poultry by-product meal and 15% H. illucens; 80PBM-20HI, 80% protein from poultry by-product meal and 20% H. illucens and 75PBM-25HI, 75% protein from poultry by-product meal and 25% H. illucens*.

a *Feed formulation and nutritional composition from our previous study (43)*.

† *Includes also C8:0, C10:0, C11:0, C13:0, C15:0, C17:0, C20:0, C21:0, C22:0, C23:0, and C24:0*.

‡ *Includes also C14:1n5, C15:1, C17:1, C22:1n9, and C24:1*.

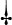
*Includes also C18:2 trans, C18:3n6, C18:4n3, C20:2, C20:3n6, C20:3n3, C22:2*.

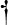
*Includes also C18:4n3 and C20:3n3*.

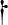
*Includes also C18:3n6, C20:3n6*.

* *Fatty acids composition of PBM and FHI from our previous study (23)*.

After acclimatization with the experimental condition, 300 barramundis with 7 g of mean initial weight were stocked into 12 tanks (73 × 84 cm and 300 L capacity) with 25 fish per tank. A biological filter, heater, and aerator were set up with each tank. Water quality parameters including dissolved oxygen, temperature, salinity, ammonia, nitrite and nitrate, and photoperiod were monitored daily and maintained within the recommended ranges as illustrated in our earlier study (22, 43). Each diet was assigned in triplicate and the fish were hand-fed twice a day (9.00 and 18.00) until satiety for 56 days. After 56 days of the feeding trial, the fish were not fed for 24 h and 8 fish/replicate were fileted immediately after stabbing the brain. Skinless filets from two fish were freeze-dried and stored at −80°C for further fatty acid analysis. For the remaining six fish, one side of the filets was transported immediately post-mortem on sterilized ice to a simulated display refrigerator for shelf-life study, while the other filets were subjected to individual quick freezing (IQF) with liquid nitrogen and stored at −80°C to preserve sensory quality until sensory evaluation.

### Proximate Composition Analysis

The crude protein, crude fat, ash, and moisture were analyzed following the Association of Official Analytical Chemists (AOAC) (44) standard methods. For protein analysis, 1 g of the sample, a boiling chip, and a Kjeldhal catalyst tablet were placed in a digestion tube. Then, 8 ml of a sulfuric acid-phosphoric acid mixture along with 4 ml of 35% hydrogen peroxide were added to the tube, and the digestion was completed using a Kjeltec digester block (Foss Tecator 2020, Högänas, Sweden) at 420°C. After distilling the digested samples using the Kjeltec distilling unit (Foss Tecator 1002, Högänas, Sweden), the distillate was collected into a flask with 25 ml of boric acid indicator containing bromocresol green and methyl red which was subjected to titration with hydrochloric acid and conversion factor of 6.25 was used to calculate the crude protein content.

The crude fat content was determined using the petroleum ether extraction method where the fat from the samples was extracted using the Soxhlet unit (Extraction unit E-816, BÜCHI Labortechnik AG, Flawil, Switzerland). The extracted fat was then dried at 105°C till constant weight was obtained and the crude fat percentage was calculated by dividing the weight of the extracted fat over the sample weight.

For the ash content determination, the sample was weighed before and after heating overnight at 550°C using a muffle furnace (Thermolyne muffle furnace, model 48000, Thermo Fisher Scientific Inc, Iowa, USA). The ash content was calculated by dividing the post-heating sample weight over the initial sample weight.

For moisture estimation, the sample was weighed before and after oven drying at 105°C until a constant weight was obtained. The moisture content was then calculated by dividing the post-drying weight over the pre-drying weight.

### Fatty Acid Analysis

The fatty acid of the experimental diets and whole freeze-dried filet (skinless) were analyzed following the protocol of O'Fallon et al. (45) as reported in our earlier study (28).

### Sensory Quality

All the procedure-related sensory trial was carried out in strict compliance with the Australian Code for the Responsible Conduct of Research and National Statement on Ethical Conduct in Human Research, and reviewed and approved by the Curtin University Human Research Ethics Committee (Approval Number: HRE2020-0689).

The sensory quality of the barramundi filets at the end of the 56-day feeding trial was designated and evaluated following the method described by Gedarawatte et al. (46) and Lawless and Heymann (47). Eleven persons with no allergies, smoking habits, chronic health issues, visual impairment, respiratory issues, taste disorders, pregnant condition, not breast-feeding or on long-term medication, and who consume fish at least once every fortnight, were recruited, consented, and trained according to AS 2542.1.3:2014 (48) and CAC-GL 31-1999 (49). Before starting the sensory evaluation, screening on sensory sensitivity was conducted using multiple fish filets of various qualities. Three of the potential panelists were excluded due to their low precision in determining fish quality after repeat exposures. After that, 9 participants in the age of 18–50, consisting of 5 females and 4 males were screened and considered to be eligible and included in the study as semi-trained panelists. A labeled magnitude scale (50) was used to evaluate the visual appearance, odor, and overall acceptability of the raw barramundi samples in order to understand the quality of the barramundi in retail display.

Then, the same samples were sous-vided at 74°C for 6 min in vacuum-sealed boil-in pouches and served to the panelists within 15 min to evaluate the appearance, odor, texture, taste, and overall acceptability of the cooked samples similar to the raw samples.

### Simulated Retail Display for Shelf-Life Studies

The freshly fileted fish samples were immediately placed on ice inside an uncovered polystyrene box. The box was then placed in a 4°C refrigerator to simulate conditions in retail display. Melted ice was drained daily with new ice replaced. Six filets from different fishes were analyzed per treatment during each day on Days 1, 4, and 8 post-fileting.

#### Physical Parameters

##### Texture Profile Analysis

Before texture analysis, the fish filets were tempered at 24.5°C for 30 min. For each quality, A 5 × 5 cm fish cube sampled between the pelvic and anal fin portion along the lateral line including the dorsal and ventral portion, was then compressed using the texture analyzer TVT 6700 (PerkinElmer, Inc., Waltham, Middx, USA) equipped with a 20 kg load cell and a 25 mm flat-ended cylindrical probe. Two consecutive cycles of 50% compression with 5 s in between were conducted under a constant speed of 50 mm/min. Six texture parameters: hardness, cohesiveness, adhesiveness, springiness, gumminess, and chewiness were obtained from each analysis using Bourne's (51) calculation methods and the TexCal 5.0 instrumental software.

##### Microscopic Observation of Filet Tissues

One portion of muscle at days 1, 4, and 8 stored at 0°C was cut from three filets/treatment and immediately fixed in 10% buffered formalin before dehydrating with a series of alcohol. Then, the samples were embedded in paraffin, sectioned to 5 μm thickness, and stained with hematoxylin and Eosin (H&E) for observation under a light microscope according to the standard histological procedure.

##### Drip Loss

Drip loss was calculated from day 0 to 8 by dividing the weight loss over the initial weight of the fresh fish samples and expressed as a percentage.

##### Color

Prior to color measurement, HunterLab ColorFlex colorimeter (Hunter Association Laboratory Inc., Reston, VA, USA) was calibrated using manufacturer standards. Surface color coordinates (L^*^, a^*^, b^*^) were then obtained from portions along the lateral filet line.

##### Quality Index

The QI of the barramundi filets was determined following the method described by Fuentes-Amaya et al. (52). In brief, the skin, appearance, and flesh of the fish were scored out of 10 based on quality parameters such as brightness, transparency, texture, blood color, odor, and gaping.

#### Chemical Parameters

##### pH

Approximately 1 g of muscle tissue was homogenized with 10 ml of distilled water, mixed using a rotary suspension mixer (Ratek, Boronia, Vic, AU) for 30 min and the pH values of the aliquot were determined using a three-scale calibrated Aqua-pH meter (TPS Pty, Ltd., Brendale, QLD, AU) (44).

##### Lipid Oxidation

Lipid oxidation was determined using 2-thiobarbituric acid reactive substances (TBARS) following the method of Raharjo et al. (53) with some modifications. A total of 10 g of muscle tissue were added to 40 ml of 5% trichloroacetic acid (TCA) along with 1 ml of 0.15% 2,6-di-teri-butil-4-methylphenol (BHT) in ethanol. The mixture was homogenized, filtered, and adjusted with 5% TCA to 50 ml. After which, 2 ml of 0.08 M 2-thiobarbutric acid (TBA) was added to 2 ml of sample in a screw cap test tube, heated at 100°C for 10 min. Absorbance was then measured at 532 nm and concentration was calculated using standards prepared with 1,1,3,3-tetrathyoxypropane (TEP) in 20% TAC at 1–10 μM.

#### Statistical Analysis

All results are presented as mean ± SE. To determine the effect of diet and storage, two-way ANOVA was performed with Tukey's multiple comparison test if the data met normality and homogeneity of variance, checked by Kolmogorov-Smirnov and Levene's tests, respectively. If any of the factors were significant, one-way ANOVA was performed individually to compare the data among the diets and storage time. Data on proximate composition and fatty acid were compared using one-way ANOVA with Tukey's multiple comparison test. Variations were considered as significant at 0.05 < *P* < 0.001.

## Results

### Filet Proximate Composition and Fatty Acid Profile

The proximate composition of barramundi filet fed PBM-HI was similar to the 0PBM-0HI fed barramundi filet, except for crude lipid which increased in the filet of the barramundi fed with PBM-HI based diets (Table 2). The total saturated fatty acid (SFA) increased significantly due to a gradual increase in C12:0 (lauric acid) and C14:0 (myristic acid) in the filet of the barramundi fed with PBM-HI-based diets. A gradual increase of HI larvae meal in the diets also elevated the level C12:0 and C14:0 in the filets from the respective diets. An elevation of the C16:1n7 and C18:1cis + trans concentration resulted in an increase in the total concentration of monounsaturated fatty acid (MUFA) in the filet of the barramundi fed with PBM-HI-based diets. The lower concentration of total n-3 polyunsaturated fatty acid (PUFA) and n-6 PUFA, particularly, C20:5n3, C22:5n3, C22:6n3, C20:4n6, and C22:4n6, decreased the total PUFA in PBM-HI fed barramundi filets. A similar tendency was observed in the respective diets (Table 1).

**Table 2:** Filet proximate composition and fatty acid profile (% of total FA) of barramundi (*n* = 6) fed with the mixture of PBM and full-fat black soldier fly larvae.

	**Experimental diets**			
	**0PBM-0HI**	**85PBM-15HI**	**80PBM-20HI**	**75PBM-25HI**
**Proximate composition (%, Wet basis)**				
Moisture	76.10 ± 0.25	76.23 ± 0.35	75.57 ± 0.05	76.40 ± 0.35
Crude protein	20.58 ± 0.56	20.04 ± 0.24	20.23 ± 0.27	20.38 ± 0.39
Crude lipid	1.77 ± 0.15^b^	2.04 ± 0.35^ab^	2.25 ± 0.05^a^	2.86 ± 0.61^ab^
Ash	1.10 ± 0.07	1.12 ± 0.05	1.17 ± 0.03	1.07 ± 0.02
**Fatty acid (% of total fatty acid)**				
C12:0	1.39 ± 0.84^c^	4.43 ± 0.16^b^	6.67 ± 0.14^a^	8.65 ± 0.22^a^
C14:0	2.30 ± 0.14^c^	2.28 ± 0.03^c^	2.87 ± 0.04^b^	3.47 ± 0.06^a^
C16:0	19.26 ± 0.24^a^	17.51 ± 0.08^b^	17.60 ± 0.29^b^	17.69 ± 0.02^b^
C16:1n7	3.35 ± 0.15^c^	3.64 ± 0.05^bc^	3.94 ± 0.04^ab^	4.17 ± 0.04^a^
C18:0	6.39 ± 0.13^a^	5.51 ± 0.12^b^	5.21 ± 0.10^b^	5.25 ± 0.03^b^
C18:1cis + trans	25.53 ± 1.57^b^	36.64 ± 0.11^a^	35.05 ± 0.17^a^	33.32 ± 0.10^a^
C18:2 cis	9.59 ± 0.81^b^	15.44 ± 0.09^a^	14.72 ± 0.03^a^	14.33 ± 0.14^a^
C18:3n3	2.00 ± 0.12^c^	3.33 ± 0.06^a^	3.21 ± 0.44^ab^	3.00 ± 0.02^b^
C20:1	1.20 ± 0.03^b^	1.38 ± 0.01^a^	1.40 ± 0.00^a^	1.13 ± 0.02^b^
C20:4n6	1.93 ± 0.06^a^	1.48 ± 0.09^b^	1.31 ± 0.05^b^	1.31 ± 0.08^b^
C20:5n3	2.51 ± 0.20^a^	1.22 ± 0.03^b^	1.35 ± 0.04^b^	1.30 ± 0.06^b^
C22:4n6	1.58 ± 0.24^a^	0.16 ± 0.01^b^	0.14 ± 0.01^b^	0.14 ± 0.01^b^
C22:5n3	1.71 ± 0.09^a^	0.98 ± 0.05^b^	0.95 ± 0.04^b^	0.95 ± 0.03^b^
C22:6n3	17.38 ± 2.66^a^	2.99 ± 0.18^b^	2.52 ± 0.14^b^	1.86 ± 0.10^b^
SFA^†^	31.20 ± 0.56^b^	30.73 ± 0.18^b^	33.40 ± 0.31^a^	36.20 ± 0.32^a^
MUFA^‡^	30.57 ± 1.64^b^	42.03 ± 0.13^a^	40.77 ± 0.20^a^	39.00 ± 0.12^a^
PUFA[Table-fn TN10]	38.37 ± 2.20^a^	27.37 ± 0.23^b^	25.97 ± 0.35^b^	24.93 ± 0.35^b^
n-3 PUFA[Table-fn TN11]	24.10 ± 2.81^a^	9.03 ± 0.18^b^	8.63 ± 0.23^b^	7.77 ± 0.17^b^
n-6 PUFA[Table-fn TN12]	4.27 ± 0.19^a^	2.60 ± 0.06^b^	2.30 ± 0.12^b^	2.53 ± 0.09^b^

*Different superscript letters in the same row indicate significant differences while the mean holding no superscript letters indicates no variation between fishmeal (FM) based diet (0PBM-0HI) and test diets*.

† *Includes also C8:0, C10:0, C11:0, C13:0, C15:0, C17:0, C20:0, C21:0, C22:0, C23:0, and C24:0*.

‡ *Includes also C14:1n5, C15:1, C17:1, C22:1n9, and C24:1*.

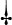
*Includes also C18:2 trans, C18:3n6, C18:4n3, C20:2, C20:3n6, C20:3n3, C22:2*.

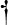
*Includes also C18:4n3 and C20:3n3*.

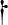
*Includes also C18:3n6, C20:3n6*.

### Sensory Attributes

The sensory evaluation of the raw and cooked flesh of the barramundi fed with fishmeal-free diets containing the different proportions of PBM and HI larvae meal for 56 days is presented in Table 3. PBM-HI-based diets improved the raw filet of barramundi, manifested by the higher scores given by the panelists for visual appearance, odor, and overall quality. Compared with 0PBM-0HI, the cooked odor was better in the filet of the barramundi fed with PBM-HI-based diets. The cooked texture and taste were unchanged by the test diets, whereas the cooked overall quality was better in the filet of the barramundi fed with PBM-HI-based diets when compared with the filet of the 0PBM-0HI fed barramundi.

**Table 3:** Sensory quality of the raw and cooked filet of barramundi fed with PBM and HI larvae meal replacing FM entirely for 8 weeks.

	**0PBM-0HI**	**85PBM-15HI**	**80PBM-20HI**	**75PBM-25HI**
Raw visual appearance	3.64 ± 0.41^b^	6.01 ± 0.65^a^	5.07 ± 0.48^ab^	6.55 ± 0.55^a^
Raw odor	3.56 ± 0.29^b^	6.60 ± 0.46^a^	5.63 ± 0.60^a^	6.37 ± 0.71^a^
Raw overall quality	3.67 ± 0.37^b^	6.04 ± 0.63^a^	5.46 ± 0.34^ab^	5.99 ± 0.62^a^
Cooked visual appearance	3.93 ± 0.58^b^	5.19 ± 0.48^ab^	6.24 ± 0.46^a^	5.99 ± 0.71^ab^
Cooked odor	3.55 ± 0.68^b^	6.14 ± 0.49^a^	5.77 ± 0.54^a^	6.23 ± 0.49^a^
Cooked texture	4.90 ± 0.61	5.24 ± 0.31	5.94 ± 0.61	6.02 ± 0.69
Cooked taste	3.99 ± 0.76	4.81 ± 0.73	5.39 ± 0.58	5.94 ± 0.64
Cooked overall quality	3.48 ± 0.72^b^	5.49 ± 0.82^ab^	5.57 ± 0.49^ab^	6.23 ± 0.61^a^

*Means are average of six biological replicates ± SE. Different superscript letters in the same row indicate significant differences while the mean holding no superscript letters indicates no variation between 0PBM-0HI and test diets. Means were compared by one-way ANOVA with Dunnett's multiple comparisons test at 0.05 < P < 0.001*.

### Drip Loss

**Figure 1:**
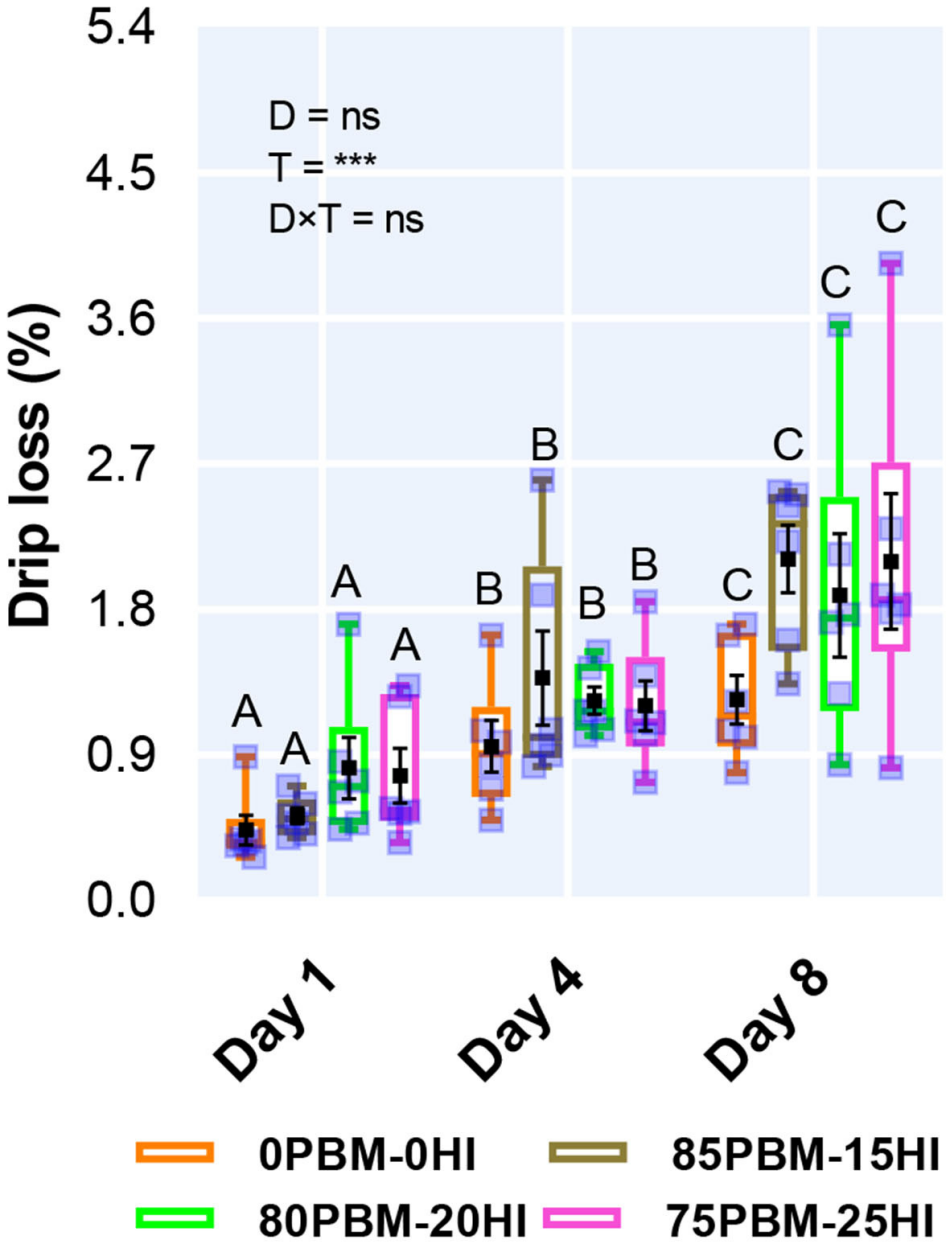
Drip loss of barramundi filet after 56 days of feeding PBM and HI-based diets during chilled storage. Box and whiskers plot indicates the range and square shape marker indicates the number of biological replicates used for the analysis. The black square shape backmarker denotes the mean with standard error. The effect of factors including diet (D) and storage time (T) and their interaction (D × T) was conducted by two-way ANOVA at *P* < 0.001. Upper case letters on the top of the box plot indicate a significant difference between storage times in all test diets.

Diet had no influence on drip loss while storage time had a significant effect on drip loss (Figure 1). The percent of drip loss increased significantly over the storage time irrespective of diet. There was no interaction between diet and storage time.

### Texture and Color of Barramundi Filet

**Figure 2:**
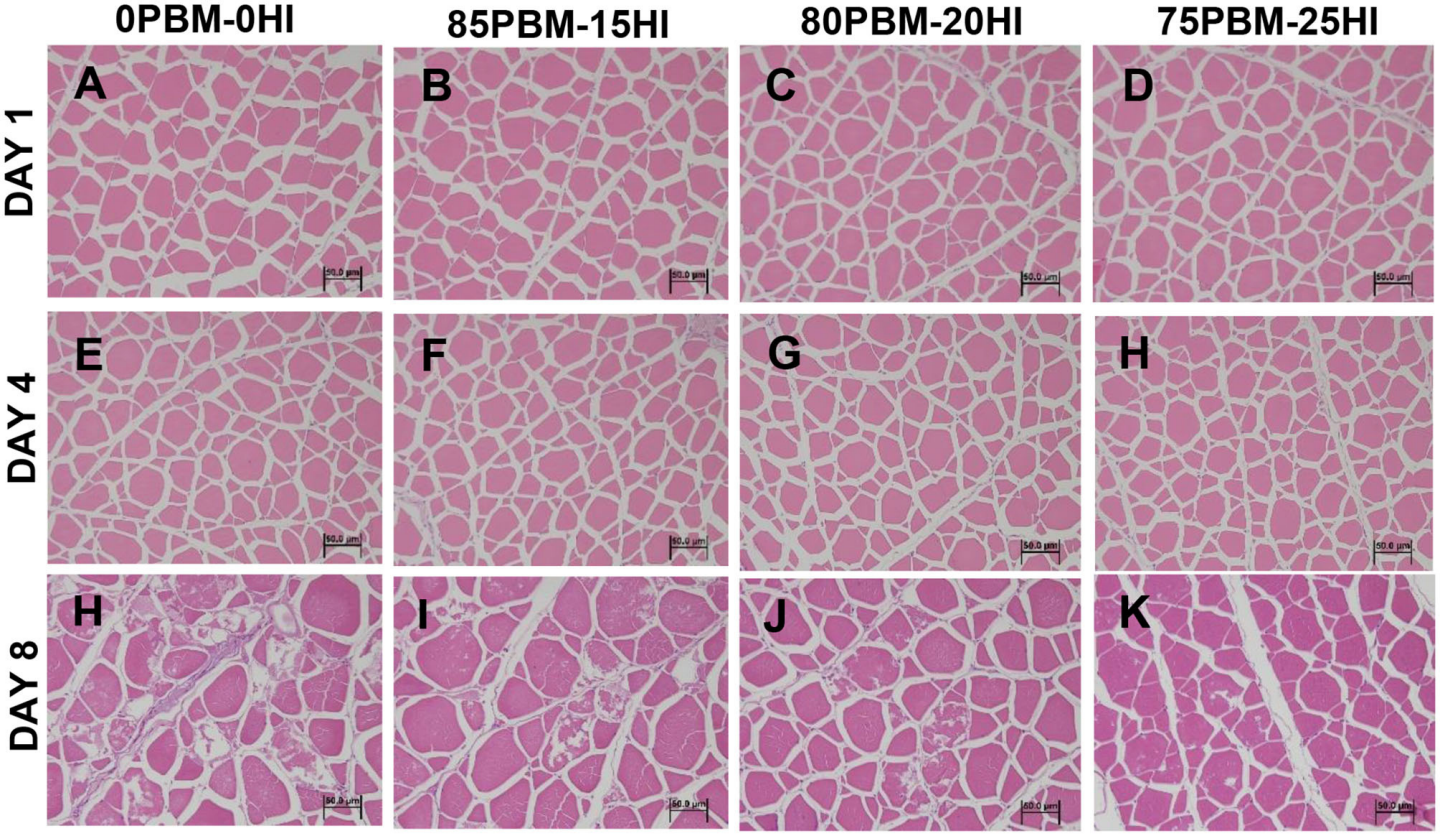
Microstructure changes in barramundi muscle (H&E staining and 40 × magnification) when fed PBM-HI based diets during the chilled storage of days 1 **(A–D)**, 4 **(E–H)**, and 8 **(I–L)**.

None of the texture profiles were influenced by the diet whereas storage time significantly influenced the cohesiveness, gumminess, and chewiness, as demonstrated by two-way ANOVA analysis (Table 4). Cohesiveness, gumminess, and chewiness decreased in all test diets over the storage time. There was no interaction found between diet and storage time in the texture profiles. Skin brightness and redness were significantly affected by diet. The brightness in the skin of barramundi fed with 85PBM-15HI and 80PBM-20HI decreased significantly on days 1 and 8. On Day 1, skin redness changed significantly in barramundi when fed with PBM-HI-based diets. Regardless of diet, skin redness over the 8 days storage time decreased. A similar change in the yellowness and chroma of the skin was found over the storage time, though diet did not change the skin yellowness and chroma. Filet brightness in response to test diets and storage time increased while flesh redness was not affected by diet and storage time. Flesh yellowness was increased by diet and storage time. Skin chroma was unaffected by diet and storage time. The microstructure of the muscle tissues in response to diets and over the storage time is presented in Figure 2. On Days 1 (Figures 2A–D) and 4 (Figures 2E–H), the muscle micro-structure was unchanged, supported by the tight attachment and regular shape of myofibrils together with a uniform distribution and distinct connective tissues. However, on day 8, the muscle tissue of the barramundi fed with 0PBM-0HI (Figure 2I) showed muscle degeneration and atrophy while the barramundi fed with PBM-HI-based diets (Figures 2J–L) demonstrated mild structural changes in muscles with 75PBM-25HI showing the least changes. It is also worth noting that an increase in intermyofibrillar spaces, reduced fiber-fiber adhesion, ruptured fiber, and loss of cell borders were observed on day 8.

**Table 4:** Texture and color changes of barramundi fed control and test diets containing different proportions of PBM and HI larvae meal during chilled storage.

		**Experimental diets**				**Two-way ANOVA**		
		**0PBM-0HI**	**85PBM-15HI**	**80PBM-20HI**	**75PBM-25HI**	**D**	**T**	**D × T**
**Texture**								
Springiness (mm)	Day 1	0.99 ± 0.01	0.99 ± 0.00	0.99 ± 0.01	0.99 ± 0.00			
	Day 4	0.99 ± 0.00	0.99 ± 0.00	0.99 ± 0.00	0.99 ± 0.00	0.62	0.66	0.89
	Day 8	0.99 ± 0.00	0.99 ± 0.00	0.99 ± 0.00	0.99 ± 0.00			
Cohesiveness (ratio)	Day 1	0.33 ± 0.02	0.34 ± 0.01	0.35 ± 0.01	0.33 ± 0.01			
	Day 4	0.30 ± 0.02	0.31 ± 0.02	0.32 ± 0.01	0.31 ± 0.01	0.46	0.00	0.98
	Day 8	0.30 ± 0.00	0.30 ± 0.00	0.31 ± 0.01	0.32 ± 0.01			
Gumminess (g)	Day 1	1,673.56 ± 92.96	1,493.84 ± 88.88	1,713.28 ± 17.91	1,711.11 ± 136.02			
	Day 4	1,268.03 ± 175.40	1,375.89 ± 148.82	1,299.16 ± 93.19	1,485.75 ± 103.87	0.28	0.01	0.77
	Day 8	1,297.75 ± 51.85	1,418.23 ± 114.66	1,585.28 ± 107.74	1,568.49 ± 164.75			
Chewiness (g/mm)	Day 1	1,668.96 ± 93.86	1,492.86 ± 87.19	1,709.05 ± 175.85	1,698.33 ± 132.14			
	Day 4	1,262.16 ± 174.43	1,371.71 ± 148.42	1,293.11 ± 93.60	1,477.99 ± 102.87	0.29	0.01	0.77
	Day 8	1,292.66 ± 50.91	1,407.80 ± 113.14	1,576.33 ± 106.37	1,559.56 ± 164.11			
Adhesiveness (g/s)	Day 1	−39.60 ± 10.22	−37.39 ± 6.93	−42.00 ± 11.16	−31.18 ± 7.08			
	Day 4	−46.63 ± 15.48	−38.80 ± 5.15	−40.96 ± 9.54	−32.75 ± 7.65	0.67	0.93	0.88
	Day 8	−47.34 ± 11.17	−49.58 ± 20.25	−25.81 ± 6.27	−39.63 ± 15.15			
Hardness (g)	Day 1	5,067.50 ± 216.77	4,353.00 ± 284.39	4,830.67 ± 350.69	5,146.67 ± 354.13			
	Day 4	4,170.00 ± 341.12	4,449.67 ± 316.38	4,138.67 ± 283.77	4,721.33 ± 231.06	0.20	0.05	0.35
	Day 8	4,324.17 ± 116.64	4,663.50 ± 220.70	5,014.00 ± 229.68	4,926.33 ± 295.69			
**Skin color**								
L*	Day 1	55.97 ± 1.45	52.27 ± 0.99	50.30 ± 0.93	53.06 ± 1.82			
	Day 4	54.75 ± 1.30	52.35 ± 0.79	52.52 ± 0.51	54.06 ± 0.90	0.00	0.73	0.42
	Day 8	54.41 ± 1.14	50.07 ± 0.97	51.78 ± 0.83	54.97 ± 1.69			
a*	Day 1	−0.73 ± 0.05	−0.41 ± 0.09	−0.27 ± 0.12	−0.16 ± 0.15			
	Day 4	−0.63 ± 0.09	−0.44 ± 0.11	−0.54 ± 0.04	−0.58 ± 0.08	0.01	0.00	0.07
	Day 8	−0.77 ± 0.07	−0.50 ± 0.11	−0.69 ± 0.10	−0.73 ± 0.14			
b*	Day 1	−4.04 ± 0.72	−4.95 ± 0.46	−5.45 ± 0.36	−4.21 ± 0.34			
	Day 4	−5.39 ± 0.63	−5.31 ± 0.28	−5.32 ± 0.20	−6.03 ± 0.48	0.18	0.02	0.24
	Day 8	−4.75 ± 0.37	−5.61 ± 0.29	−5.49 ± 0.21	−4.87 ± 0.21			
C*	Day 1	4.40 ± 0.62	4.98 ± 0.45	5.47 ± 0.36	4.25 ± 0.34B			
	Day 4	5.44 ± 0.63	5.34 ± 0.28	5.61 ± 0.27	6.06 ± 0.48A	0.21	0.01	0.37
	Day 8	4.83 ± 0.36	5.65 ± 0.29	5.54 ± 0.21	4.95 ± 0.20AB			
**Flesh color**								
L*	Day 1	44.00 ± 0.98	49.37 ± 0.46	48.15 ± 0.63	45.79 ± 0.84			
	Day 4	45.74 ± 1.13	49.23 ± 0.29	49.50 ± 1.25	49.02 ± 0.37	0.00	0.00	0.02
	Day 8	46.74 ± 0.45	48.66 ± 0.62	48.55 ± 0.38	50.30 ± 0.63			
a*	Day 1	4.47 ± 1.09	1.42 ± 0.3	1.76 ± 0.31	3.41 ± 0.85			
	Day 4	2.01 ± 0.44	2.06 ± 0.24	2.84 ± 1.00	2.26 ± 0.53	0.23	0.18	0.03
	Day 8	1.80 ± 0.33	1.96 ± 0.58	2.10 ± 0.42	2.09 ± 0.26			
b*	Day 1	6.29 ± 0.63	5.68 ± 0.21	5.32 ± 0.31	5.86 ± 0.40			
	Day 4	5.15 ± 0.47	6.45 ± 0.27	7.31 ± 0.55	6.33 ± 0.51	0.00	0.06	0.00
	Day 8	4.79 ± 0.31	6.77 ± 0.44	7.29 ± 0.30	7.02 ± 0.22			
C*	Day 1	7.97 ± 1.11	5.93 ± 0.23	5.65 ± 0.39	7.05 ± 0.74			
	Day 4	5.63 ± 0.56	6.80 ± 0.32	8.10 ± 0.96	6.81 ± 0.36	0.26	0.88	0.00
	Day 8	5.18 ± 0.37	7.18 ± 0.61	7.66 ± 0.40	7.36 ± 0.26			
		**Days**			**Diets**			
		**Day 1**	**Day 4**	**Day 8**	**0PBM-0HI**	**85PBM-15HI**	**80PBM-20HI**	**75PBM-25HI**
**Two way ANOVA**								
**Texture**								
Springiness (mm)		A	A	A	a	a	a	a
Cohesiveness (ratio)		A	B	B	a	a	a	a
Gumminess (g)		A	B	AB	a	a	a	a
Chewiness (g/mm)		A	B	AB	a	a	a	a
Adhesiveness (g/s)		A	A	A	a	a	a	a
Hardness (g)		A	B	AB	a	a	a	a
**Skin color**								
L*		A	A	A	a	bc	c	ab
a*		A	AB	B	b	a	ab	a
b*		A	B	AB	a	a	a	a
C*		B	A	AB	a	a	a	a
**Flesh color**								
L*		B	A	A	b	a	a	a
a*		A	A	A				
b*		A	A	A	b	a	a	a
C*		A	A	A	a	a	a	a

The effect of factors including diet (D) and storage time (T) and their interaction (D × T) was conducted by two-way ANOVA. L*, brightness; a*, red/green; b*, yellow/blue and c*, Chroma.

### The Linear Relationship Between QI Score and Post-Days Storage

**Figure 3:**
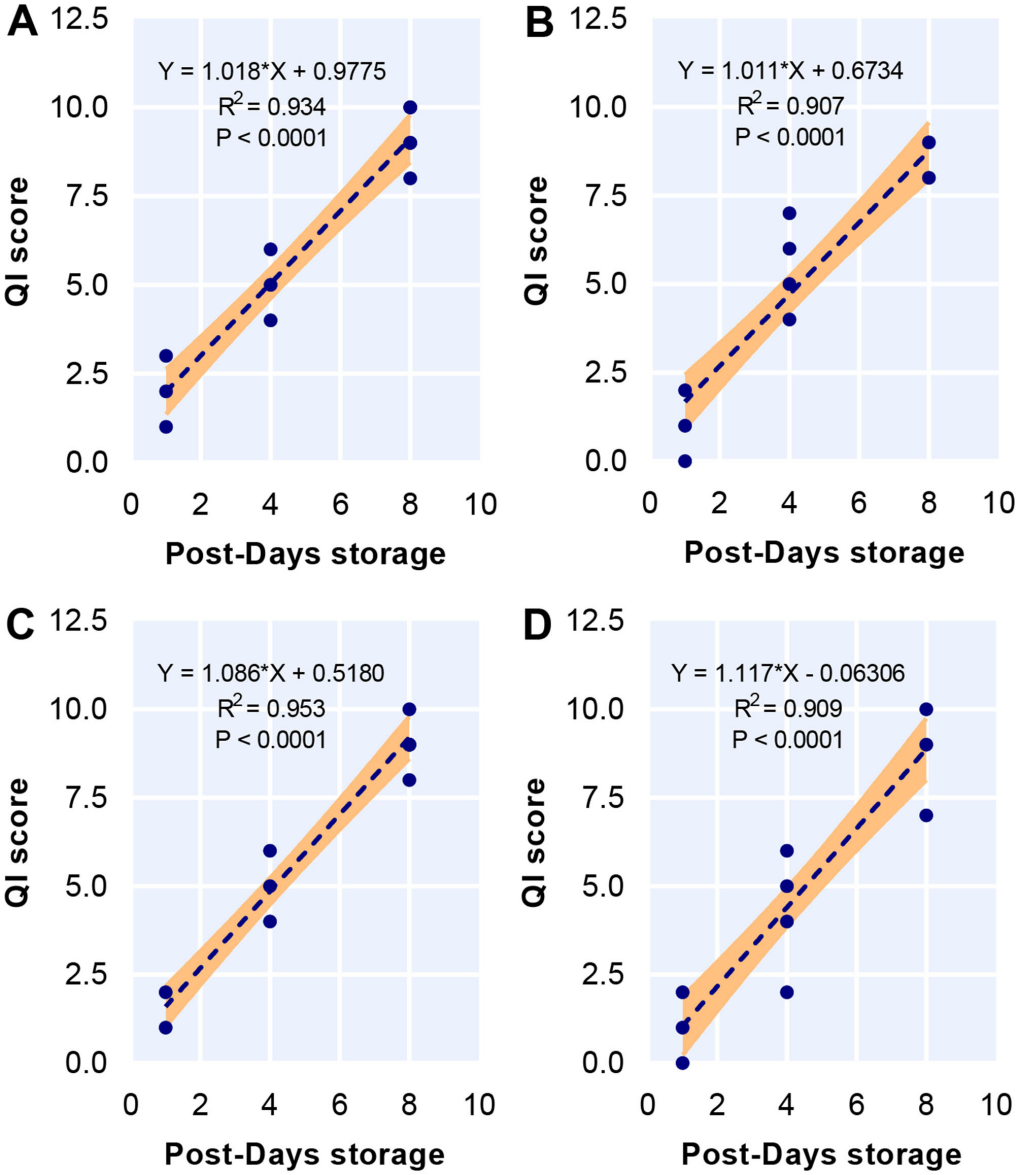
Linear correlation between quality index (QI) and storage time for test diets including 0PBM-0HI **(A)**, 85PBM-15HI **(B)**, 80PBM-20HI **(C)**, and 75PBM-25HI **(D)**.

Diet did not influence any of the attributes including skin brightness, appearance transparency, flesh texture, flesh blood, flesh odor, and flesh gaping used for QI schemes while storage time affected these attributes, as demonstrated by two-way ANOVA (Table 5). The score of all attributes increased over the storage time regardless of diet. No significant interaction was found between diet and storage time. There were a strong linear relationship between storage time and QI score in 0PBM-0HI (*R*^2^ = 0.934) (Figure 3A), 85PBM-15HI (*R*^2^ = 0.91) (Figure 3B), 80PBM-20HI (*R*^2^ = 0.91) (Figure 3C), and 75PBM-15HI (*R*^2^ = 0.91) (Figure 3D).

**Table 5:** Results of sensory parameters evaluated by quality index method (QIM): skin brightness, appearance transparency, flesh texture, flesh blood, flesh odor, and flesh gaping in barramundi filet fed with PBM-HI-based diets during chilled storage.

		**Experimental diets**				**Two-way ANOVA**		
**QI parameters**		**0PBM-0HI**	**85PBM-15HI**	**80PBM-20HI**	**75PBM-25HI**	**D**	**T**	**D × T**
Skin brightness	Day 1	-	-	-	-			
	Day 4	-	–	-	-	0.53	0.00	0.62
	Day 8	1.33 ± 0.21	1.00 ± 0.00	1.17 ± 0.17	1.17 ± 0.17			
Appearance transparency	Day 1	-	-	-	-			
	Day 4	0.50 ± 0.22	0.67 ± 0.21	0.83 ± 0.17	0.33 ± 0.21	0.44	0.00	0.32
	Day 8	1.00 ± 0.00	0.83 ± 0.17	1.00 ± 0.00	1.00 ± 0.00			
Flesh texture	Day 1	0.17 ± 0.17	0.17 ± 0.17	-	-			
	Day 4	0.83 ± 0.17	0.67 ± 0.21	0.50 ± 0.22	0.67 ± 0.21	0.48	0.00	0.92
	Day 8	1.00 ± 0.00	1.00 ± 0.00	1.00 ± 0.00	1.00 ± 0.00			
Flesh blood	Day 1	0.17 ± 0.17	-	-	-			
	Day 4	1.00 ± 0.00	1.17 ± 0.17	1.17 ± 0.17	1.00 ± 0.26	0.84	0.00	0.94
	Day 8	1.83 ± 0.17	1.67 ± 0.21	1.83 ± 0.17	1.67 ± 0.33			
Flesh odor	Day 1	-	-	-	-			
	Day 4	1.00 ± 0.00	1.00 ± 0.00	1.00 ± 0.00	1.00 ± 0.00	0.40	0.00	0.43
	Day 8	1.83 ± 0.17	2.00 ± 0.00	2.00 ± 0.00	2.00 ± 0.00			
Flesh gaping	Day 1	1.50 ± 0.22	1.17 ± 0.31	1.33 ± 0.21	1.00 ± 0.26			
	Day 4	2.00 ± 0.00	1.83 ± 0.17	1.83 ± 0.17	1.50 ± 0.22	0.14	0.00	0.78
	Day 8	2.00 ± 0.00	2.00 ± 0.00	2.00 ± 0.00	2.00 ± 0.00			
		**Day 1**	**Day 4**	**Day 8**	**0PBM-0HI**	**85PBM-15HI**	**80PBM-20HI**	**75PBM-25HI**
**Two-way ANOVA**								
Skin brightness		B	B	A	a	a	a	a
Appearance transparency		C	B	A	a	a	a	a
Flesh texture		C	B	A	a	a	a	a
Flesh blood		C	B	A	a	a	a	a
Flesh odor		C	B	A	a	a	a	a
Flesh gaping		B	A	A	a	a	a	a

*The effect of factors including diet (D) and storage time (T) and their interaction (D × T) was conducted by two-way ANOVA*.

### pH and Lipid Oxidation

**Figure 4:**
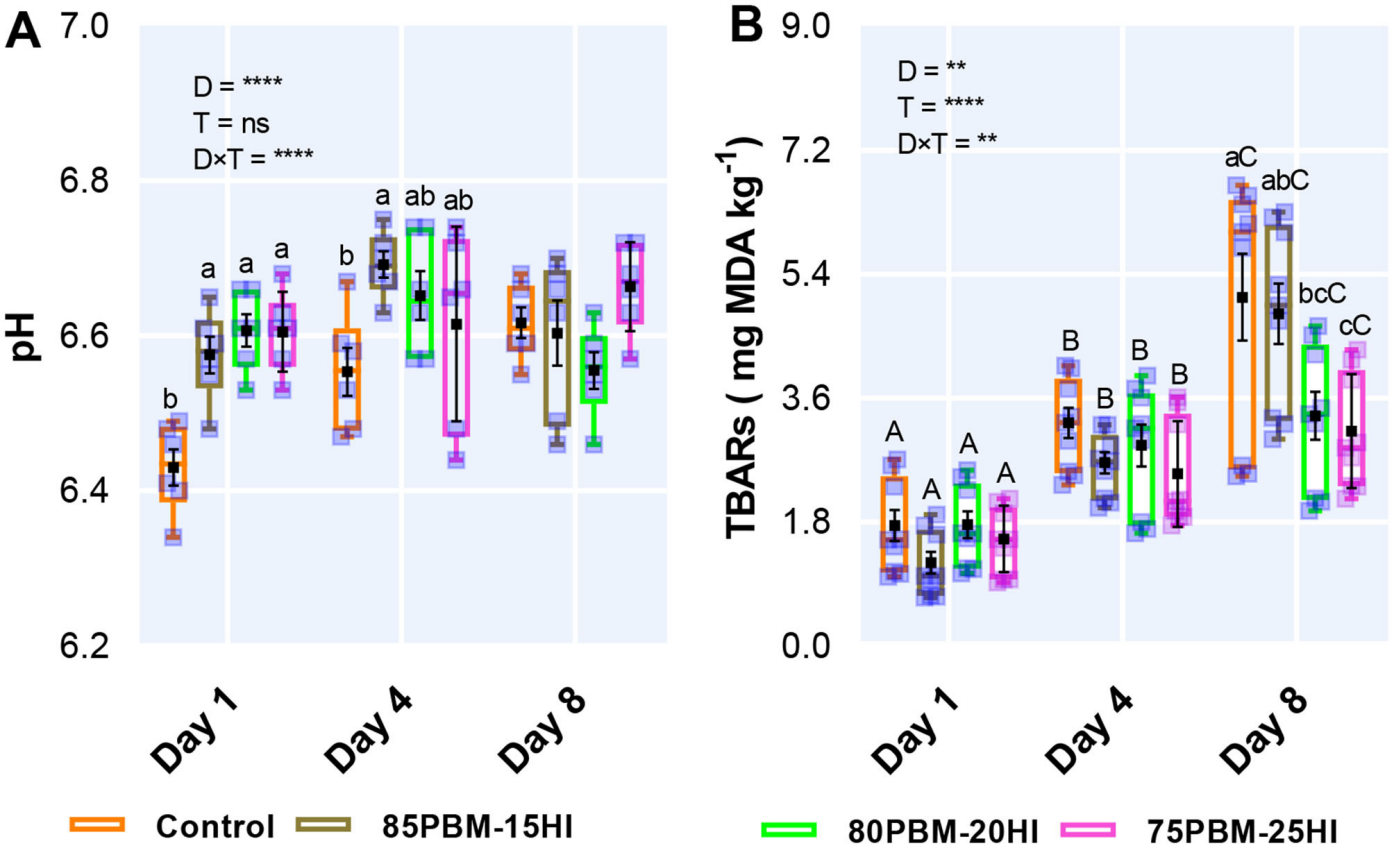
The production of pH **(A)** (*n* = 6) and rancidity **(B)** [2-thiobarbituric acid reactive substances (TBARS)] (*n* = 9) of barramundi filet after 56 days of feeding PBM and HI based diets during chilled storage. Box and whiskers plot indicates the range and square shape marker indicates the number of biological replicates used for analysis. Square shape black marker denotes the mean with standard error. The effect of factors including diet (D) and storage time (T) and their interaction (D × T) was conducted by two-way ANOVA at **P < 0.01 and ****P < 0.0001. Lower case letters on the top of the box and whisker plot denote significant differences within the treatment in each day while Upper case letters on the top of the box plot indicate a significant difference between storage times in all test diets.

The results of the two-way ANOVA of the pH and TBARs activity of barramundi filet in response to diets and storage time are presented in Figure 4. The pH of the filet was influenced by diet and showed an interaction between diet and storage time (Figure 4A). The pH of the barramundi filet upsurged at Day 1 and 4 when fed with the mixture of PBM and HI larvae meal. However, storage time had no significant effect on pH. The malondialdehyde (MDA) content, measured by TBARS, was influenced by both factors with a significant interaction (Figure 4B). On day 8, PBM-HI-based diets retarded the production of MDA in the flesh of barramundi but there was a gradual increase in lipid oxidation production over the storage time irrespective of diets.

## Discussion

The production data and other physiological responses to the different diet in terms of growth and body indices, muscle amino acid composition, gut mucosal morphology and microbiota, serum metabolites, antioxidant activity, and resistance to *Vibrio harveyi* have been illustrated in our earlier article (43). Briefly, growth performance in the test diets was comparable to the fish-fed FM-based control diet, with no changes in body indices and muscle amino acid composition. However, the distal intestine mucosal barrier functions were improved by PBM-HI-based diets, as further supported by the positive changes in the abundance of beneficial microbiota. The purpose of this study, however, was to align filet quality traits using multidisciplinary approaches to test parameters including proximate and fatty acid profile, sensory attributes, texture correlating with the muscle structure, and color and lipid oxidation of barramundi filet after 56 days of feeding on diets representing a complete replacement of FM with animal-based protein.

### Filet Proximate Composition

The crude protein levels were unaffected by the mixture of PBM and full-fat HI larvae meal, suggesting that barramundi can assimilate protein from PBM-HI as effectively as FM protein. Likewise, barramundi fed with poultry protein concentrate alone (6.7–20%) or supplemented with phosphorous (15), hybrid grouper fed blends of PBM, shrimp meal, and spray-dried blood meal (20–80%) (54) and juvenile black sea bass fed with PBM (40–100) (55) produced a similar whole-body protein content to fish fed FM protein. However, a higher lipid concentration reported here indicated the flesh quality improvement of barramundi flesh since lipid concentration has been reported to be associated with the taste and appearance of cooked flesh (56, 57). This result was mainly due to the inclusion of FHI larvae which contain a higher concentration of lipid.

Regarding the muscle FA profile, an increase in total SFA in the muscle of barramundi fed with 80PBM-20HI and 75PBM-25HI was due to the substantial increase in lauric acid (C12:0) and myristic acid (C14:0). The HI larvae meal is a rich source of C12:0 and C14:0 which have been reported to enhance the total SFA concentration in barramundi (23, 24) and other fish species such as Siberian sturgeon, *Acipenser baerii* (58), rainbow trout (59), and Eurasian perch, *Perca fluviatilis* (60). Oleic acid plus elaidic acid (C18: 1cis + trans), causing a higher concentration of MUFA in PBM-HI-based diets clearly improved the MUFA concentration of barramundi in the present study. This is similar to what has previously been reported in the MUFA concentration of barramundi (22–24, 28), juvenile coho salmon, *Oncorhynchus kisutch* (61), Atlantic Salmon, *S. salar* (62), and juvenile gilthead seabream, *Sparus aurata* (57) fed with PBM. PBM is a rich source of oleic acid, resulting in higher MUFA concentration in the aforementioned studies, however, it contains a lower concentration of n-3 LC-PUFA, EPA (20:5n-3), and DHA (22:6n-3). Similarly, a lower concentration of n-3 LC-PUFA, EPA, and DHA decreased the total PUFA in PBM-HI-based diets, perhaps underpinning the lower concentration PUFA in barramundi filet fed with PBM-HI based diets. Similarly, the high inclusion of PBM resulted in a lower concentration of PUFA in barramundi, totoaba juveniles, *Totoaba macdonaldi*, and juvenile coho salmon, *Oncorhynchus kisutch*.

### Sensory Attributes

Sensory quality is important to investigate the potentiality of new alternative protein ingredients and is associated with consumer acceptance. PBM-HI-based diets improved the raw and cooked filet quality of barramundi, perhaps suggesting that the aligned increase in filet lipid might be aligned with the improved sensory quality of the barramundi filet. Besides lipid content, chitin present in HI larvae might have played a role in improving sensory filet quality as chitosan coating has been reported to improve the sensory scores in seafood (63–66), however, the mode of actions of chitin in HI larvae meal after feeding in improving the filet quality is not well-understood. The present study provides the first insight on the improvement of barramundi filet quality when fed with FM–free diets containing a mixture of PBM and HI larvae meal. However, the total substitution of FM with PBM deteriorated the sensory quality of female tenches, *Tinca MacDonald* (26), and meat meal did not affect the sensory quality of the barramundi filet (25). Hence, further study is needed to identify the components in HI larvae meals that may improve the filet quality of fish.

### Drip Loss

Evaluation of drip loss is of importance in determining the post-harvest fish quality due to the fact that it is directly related to lower water holding capacity caused by fiber shrinkage, cell damage, lower protein solubility, and protein denaturation (67), leading to sensory loss including texture and flavor (68), loss of water-soluble nutrients such as protein and amino acids (69), and financial implications (68). The unchanged drip loss in the filet of barramundi fed PBM-HI based diets similar to the unchanged filet water holding capacity of rainbow trout fed HI larvae prepupae meal (70) and blackspot sea bream, *Pagellus bogaraveo* fed mealworm larvae meal (71). This finding is consistent with the results of texture and lipid oxidation. Regardless of diet, gradual increase in drip loss over the 8 days storage is consistent with the findings of Siah and Ariff (72) who reported an increase in drip loss in barramundi flesh samples over time during storage at 2 ± 2°C but was not consistent with the findings of Wilkinson et al. (73) who reported no differences in drip loss in barramundi flesh samples over 4 days storage at 2–4°C. The result is within expectation as degradation in structure and gradual increase in lipid oxidation was found, indicating that the integrity of cell membranes reduces with storage time, enhancing passage of sarcoplasmic fluid which lead to an increase in drip loss (74, 75). However, the changes in drip loss from 0.16 to 2.86% cannot be considered as high and therefore not a major problem in chilled barramundi, as reported by earlier studies (67, 76, 77).

### Texture Profile and Microstructure of Muscle

A good number of studies have been evaluated the efficacy of FM replacement with PBM on 33 different fish species, though none of them have been evaluated the flesh quality (20). However, our recent study found no changes in the final flesh quality of barramundi in terms of pH, texture, and color when fed PBM-based diets, concurrently supplemented with tuna hydrolysate and HI larvae meal (24). Similarly, in this study texture including springiness, cohesiveness, gumminess, chewiness, and hardness of barramundi filets were not affected by the mixture of PBM and full-fat HI larvae meal. Regardless of PBM inclusion, HI larvae meal (33–100%) had no influence on the texture (hardness, cohesiveness, resilience, and adhesiveness) of salmon (78). This is similar to what has been reported by Iaconisi et al. (71) who found no variation in filet hardness, cohesiveness, gumminess, and adhesiveness of sea bream, *Pagellus bogaraveo* fed mealworm, *Tenebrio molitor*. However, storage time had a significant effect on the cohesiveness, gumminess, chewiness, and hardness, which decreased significantly on day 8. A similar result was found in shelf-life studies conducted in sea bream, *Sparus aurata*, where identical parameters reduced over time (79, 80). This phenomenon has shown to be highly related to histological structure, especially the increase in intermyofibrillar spaces, reduced fiber-fiber adhesion, ruptured fiber, and loss of cell borders. Thus, it can be suggested that textural changes were a consequence of structural degradation over time. These results are further supported by elevated lipid oxidation over the storage time in the present study, suggesting that protein deterioration took place by the action of endogenous cathepsins and exogenous protease due to microorganisms (81, 82). Hence, whole microbiome profile evaluation using modern tool rather than plate counting in barramundi filet in response to PBM-HI based diet during shelf life study is needed to be thoroughly investigated.

### Filet Color

The Color of fish is an important factor influencing consumer preference and it is largely governed by dietary and post-harvest factors in fish since fish do not have *de novo* power to synthesis color. There is a lack of studies concerning the effect of processed animal protein on the color of fish. However, the complete replacement of FM with PBM, concurrently supplemented with HI larvae meal and tuna hydrolysates did not change skin and filet color in barramundi (24). In this study, the skin brightness (L^*^) was affected by diet and this variation in the present study could be due to light or lack of shading. Farmed fish are more exposed to light than wild fish, leading to darker skin chromophores. These suggest the farmed fish may be darker than wild fish (83). This was proven by Howieson et al. (16) who reported that barramundi reared in shaded tanks were less dark than the barramundi reared in unshaded tanks. PBM-HI-based diets improved the redness (a^*^) in barramundi skin which could be due to the presence of β-carotene in insects (84). A similar effect was observed in blackspot seabream, *Pagellus bogaraveo* skin when fed mealworm, *Tenebrio molitor* (85).

Blue-gray discoloration due to melanosis is one of the common problems in farmed barramundi filet, reducing the appeal of the raw farmed barramundi filet on display to consumers and consequently impacting sales in the retail sectors (16). The same authors fed barramundi for 6 weeks by changing the levels of the substrate (the amino acid tyrosine) or substrate competitors (the amino acid tryptophan), or limiting enzyme (tyrosinase) co-factors like copper in the experimental diets to manipulate the melanin synthesis pathway, and however, none of these diets improved the graying issue. Irrespective of storage time, the improvement in barramundi filet brightness when fed with PBM-HI in the present study suggested that HI larvae meal might have properties to reduce melanosis. This is not similar to the report of Bruni et al. (70) and Moutinho et al. (86) who found no differences in the filet brightness of European seabass and rainbow trout fed with HI prepupae meal. However, brightness over the 8 days storage time increased irrespective of diet which is similar to the brightness of barramundi flesh over the 14 days of storage time (12). Also, the brightening of fish flesh over the prolonged storage time has been frequently reported by many studies (87–90). It could be due to the elevated lipid oxidation during the storage time which has been reported to affect the flesh color of fish. Flesh brightening may also be attributed to some other factors including alterations in light scattering properties in muscle during rigor (90, 91) and light-absorbing and reflecting properties due to decreases in the translucency of fish muscle (92, 93). Riboflavin (vitamin B2), a yellow-colored pigment variably found in most edible insects (71), may also influence the skin and flesh color of fish. The improvement in yellowness (b^*^) in the flesh of barramundi fed with PBM-HI-based diets could be due to the presence of 2.2 mg/100 g riboflavin in the HI larvae meal (94). In contrast, a 6-week feeding trial on barramundi fed with PBM-based diets supplemented with 5 and/or 10% of HI larvae and tuna hydrolysate did not influence the skin and flesh yellowness (24). A similar influence to a previous study on the yellowness of European seabass, *Dicentrarchus labrax* juveniles filets was found when fed pre-pupae larvae (86). These discrepancies in color variation might also be attributed to different inclusion levels of HI larvae meal, trial duration, and different fish species.

### QI

Quality index (QI) is a scoring system to estimate the freshness and quality of fishery products based on some attributes including appearance, odor, and texture changing during the storage time. The score for all attributes increased gradually during the storage time but the score was below 3 (low freshness) (52) for all attributes, indicating that barramundi filets were acceptable until day 8. However, a significant linear relationship between QI score and storage time was expected since a similar trend has been reported in many fish species (95–100). After 8 days of storage, the highest demerit score for QI for all diets was similar to the results of textural attributes, muscle microstructure, and lipid oxidation in the present study.

The final product quality deterioration of fish including color degradation, muscle gaping, blood spotting, flesh texture alterations, and drip loss is associated with the reduction of pH (12, 73, 101). An elevated level of pH in the filet of barramundi fed with PBM-HI based diet in the present study is similar to what has been reported by Moutinho et al. (86) in which pH increased in the filet of European seabass fed pre-pupae meal after slaughtering and 3 days post-storage. However, pH over the storage time in the present study changed from 6.34 to 6.74 was within the normal pH range of barramundi muscle (73).

### pH and Lipid Oxidation

Although pH elevation may be attributed to the formation of nitrogenous compounds such as ammonia, dimethylamine, trimethylamine, histamine, etc., produced by endogenous enzymes and microbial enzymatic actions (102), the increase in pH at certain levels has been reported to prevent the production of volatile bases, leading bacteria to take energy from the oxidative product rather than glycogen and other normal substrates (12). Hence, an inverse association between pH and lipid has been reported in barramundi filet after 5 months of feeding of alpha-tocopherol acetate and following a 2-week post-storage time (12). A similar association was found between pH and TBARS analysis in the present study. TBARS is widely used to quantify the degree of second stage lipid peroxidation of fish that produce aldehydes and ketones from the degradation of polyunsaturated fatty acids, associated with unpleasant odor (103). PBM-HI-based diets inhibited the rancidity production (MDA) compared with 0PBM-HI in the current study, the reduction in MDA level could be related to elevated MCFAs level as it reduced the amount of double bond which is prone to be attacked by an oxidant (104). A similar trend was also supported by Table 2 fatty acid profile, where a decrease in TBARS was only found when the MCFAs were significantly higher. A previous study has suggested that HI larvae may possess antioxidant properties to halt lipid oxidation. For example, Moutinho et al. (86) reported the efficacy of HI larvae meal to reduce the production of TBARs in the filet of European seabass. The less production of TBARS in the present study might be attributed to the lower proportion of PUFA, in particular, EPA and DHA which are sensitive to peroxidation in contact with oxygen (105).

Or, the presence of chitin in HI larvae might have retarded oxidation since the deacetylated form of chitin is known as chitosan which has been reported to delay lipid oxidation by binding the free amino groups and hydroxyl radicals of the polymer with the metal ions (Fe^2+^) and free radicals on food, thus making stable macromolecular structures (66, 106, 107). Another potential reason behind these improvements in rancidity is associated with the interaction between positively charged (${\text{NH}}_{3}^{+}$) amino groups of chitosan with the negative carboxyl groups (COO^−^) situated on the outer part of the membrane or the cell wall of bacteria and fungi (66), which change the permeability of the membrane of by blocking the passage of nutrients and oxygen important for cellular metabolism, leading to cell death (108, 109). Although chitinolytic enzymes, chitinoclastic bacteria, and the assimilation of chitosan have not been reported previously in barramundi, endogenous production of chitinase has been previously reported in marine carnivorous teleost fish (110, 111). Regardless of the diets, the flesh TBARS increase over the storage time in the current study is consistent with the reported TBARS production of barramundi flesh during 2-weeks chilled storage (12) and in filets of European seabass when fed with different levels of HI pre-pupae larvae meal (86). The range (0.70–6.69 mg MDA/kg) of TBARs production in the present study up to Day 8 was below the reported critical limits (7–8 mg MDA/kg) for fish (112, 113).

## Conclusion

The complete replacement of FM with a mixture of PBM and full-fat HI larvae meal increased the lipid content while reducing the synthesis of essential fatty acid content. Interestingly, sensory quality in both raw and cooked filet was improved by PBM-HI-based diets. Drip loss in response to diet and storage time was within the acceptable range. PBM-HI-based diets enhanced the brightness, redness, and yellowness in skin and flesh, though diet did not influence the texture profile. A significant correlation between storage time and QI was observed for all diets. Test diets influenced the pH and reduced the production of rancidity over the 8 days storage time. Altogether, the mixture of PBM and HI could increase consumer acceptability and resolve the blue-grayish coloration problem in farmed barramundi. However, further research is needed to better understand the complementary effect of HI larvae functional molecules in improving the filet quality. Also, enriching HI larvae with n-3 PUFA by changing feeding substrate could be recommended to increase the synthesis of n-3 PUFA in the flesh of barramundi.

## Data Availability Statement

The original contributions presented in the study are included in the article/supplementary material, further inquiries can be directed to the corresponding author.

## Ethics Statement

The studies involving human participants were reviewed and approved by Curtin University Human Research Ethic Committee (Approval Number: HRE2020-0689). The patients/participants provided their written informed consent to participate in this study. The animal study was reviewed and approved by Curtin University Animal Ethics Committee (ARE2018-37).

## Author Contributions

MC: conceptualization, methodology, formal analysis, investigation, data curation, writing—original draft, and visualization. WC: formal analysis, methodology, and writing—review and editing. JH: supervision, writing—review and editing, and project administration. RF: supervision, conceptualization, and writing—review and editing. All authors contributed to the article and approved the submitted version.

## Funding

This research was supported by an Australian Government Research Training Program (RTP) Scholarship.

## Conflict of Interest

The authors declare that the research was conducted in the absence of any commercial or financial relationships that could be construed as a potential conflict of interest.

## Publisher's Note

All claims expressed in this article are solely those of the authors and do not necessarily represent those of their affiliated organizations, or those of the publisher, the editors and the reviewers. Any product that may be evaluated in this article, or claim that may be made by its manufacturer, is not guaranteed or endorsed by the publisher.

## References

[1] Troell M Naylor RL Metian M Beveridge M Tyedmers PH Folke C. Does aquaculture add resilience to the global food system? Proc Nat Acad Sci. (2014) 111:13257–63. 10.1073/pnas.1404067111 25136111 PMC4169979

[2] Van Vo B Bui DP Nguyen HQ Fotedar R. Optimized fermented lupin (*Lupinus angustifolius*) inclusion in juvenile barramundi (*Lates calcarifer*) diets. Aquaculture. (2015) 444:62–9. 10.1016/j.aquaculture.2015.03.019

[3] Ilham SM Fotedar R. Effects of organic selenium supplementation on growth, accumulation, haematology and histopathology of juvenile barramundi (*Lates calcarifer*) fed high soybean meal diets. Biol Trace Element Res. (2016) 174:436–47. 10.1007/s12011-016-0708-1 27106539

[4] Ilham I Fotedar R. Growth, enzymatic glutathione peroxidase activity and biochemical status of juvenile barramundi (*Lates calcarifer*) fed dietary fermented soybean meal and organic selenium. Fish Physiol Biochem. (2017) 43:775–90. 10.1007/s10695-016-0331-2 28028742

[5] Ilham I Hapsari F Fotedar R. Growth, enzymatic glutathione peroxidase activity and biochemical status of juvenile barramundi (*Lates calcarifer*) fed dietary fermented lupin meal supplemented with organic selenium. Aquac Res. (2018) 49:151–64. 10.1111/are.13444

[6] Ilham FR Munilkumar S. Effects of organic selenium supplementation on growth, glutathione peroxidase activity and histopathology in juvenile barramundi (*Lates calcarifer* Bloch 1970) fed high lupin meal-based diets. Aquaculture. (2016) 457:15–23. 10.1016/j.aquaculture.2016.02.003

[7] Van Vo B Siddik MA Fotedar R Chaklader MR Foysal MJ Pham HD. Digestibility and water quality investigations on the processed peanut (*Arachis hypogaea*) meal fed barramundi (*Lates calcarifer*) at various inclusion levels. Aquacul Rep. (2020) 18:100474. 10.1016/j.aqrep.2020.100474

[8] Van Vo B Siddik MA Fotedar R Chaklader MR Hanif MA Foysal MJ. Progressive replacement of fishmeal by raw and enzyme-treated alga, *Spirulina platensis* influences growth, intestinal micromorphology and stress response in juvenile barramundi, *Lates calcarifer*. Aquaculture. (2020) 529:735741. 10.1016/j.aquaculture.2020.735741

[9] Vo BV Siddik MA Chaklader MR Fotedar R Nahar A Foysal MJ. Growth and health of juvenile barramundi (*Lates calcarifer*) challenged with DO hypoxia after feeding various inclusions of germinated, fermented and untreated peanut meals. PLoS ONE. (2020) 15:e0232278. 10.1371/journal.pone.0232278 32352997 PMC7192418

[10] FAO. The State of World Fisheries and Aquaculture. Opportunities and Challenges. Rome: Food and Agriculture Organization of the United Nations (2012).

[11] Skirtun M Sahlqvist P Vieira S. Australian Fisheries Statistics 2012, FRDC Project 2010/208. Canberra: Australian Bureau of Agricultural and Resource Economics and Sciences (2014).

[12] Jones BC Carton AG. Effects of dietary enrichment with alpha-tocopherol acetate and post-harvest filleting on lipid oxidation and flesh quality of tropical farmed barramundi (*Lates calcarifer*). Aquaculture. (2015) 448:280–7. 10.1016/j.aquaculture.2015.05.042

[13] Williams KC Barlow CG Rodgers LJ Ruscoe I. Potential of meat meal to replace fish meal in extruded dry diets for barramundi, *Lates calcarifer* (Bloch). I Growth performance. Aquacult Res. (2003) 34:23–32. 10.1046/j.1365-2109.2003.00785.x

[14] Glencross B Blyth D Irvin S Bourne N Campet M Boisot P. An evaluation of the complete replacement of both fishmeal and fish oil in diets for juvenile Asian seabass, *Lates calcarifer*. Aquaculture. (2016) 451:298–309. 10.1016/j.aquaculture.2015.09.012

[15] Simon CJ Salini MJ Irvin S Blyth D Bourne N Smullen R. The effect of poultry protein concentrate and phosphorus supplementation on growth, digestibility and nutrient retention efficiency in barramundi *Lates calcarifer*. Aquaculture. (2019) 498:305–14. 10.1016/j.aquaculture.2018.08.069

[16] Howieson J Glencross B Little S Bourne N Aris A Partridge GJ. Understanding and Minimising “Greying” of Farmed Barramundi Fillets. Bedford Park: The Australian Seafood Cooperative Research Centre (2013).

[17] Klinger D Naylor R. Searching for solutions in aquaculture: charting a sustainable course. Annu Rev Environ Resour. (2012) 37:247–76. 10.1146/annurev-environ-021111-161531

[18] Cruz-Suárez LE Nieto-López M Guajardo-Barbosa C Tapia-Salazar M Scholz U Ricque-Marie D. Replacement of fish meal with poultry by-product meal in practical diets for *Litopenaeus vannamei*, and digestibility of the tested ingredients and diets. Aquaculture. (2007) 272:466–76. 10.1016/j.aquaculture.2007.04.084

[19] Gunben EM Senoo S Yong A Shapawi R. High potential of poultry by-product meal as a main protein source in the formulated feeds for a commonly cultured grouper in Malaysia (*Epinephelus fuscoguttatus*). Sains Malays. (2014) 43:399–405. Available online at: http://journalarticle.ukm.my/6925/2/09_Esther.pdf

[20] Galkanda-Arachchige HS Wilson AE Davis DA. Success of fishmeal replacement through poultry by-product meal in aquaculture feed formulations: a meta-analysis. Rev Aquacult. (2019) 12:1624–36. 10.1111/raq.12401

[21] Chaklader MR Siddik MAB Fotedar R Howieson J. Insect larvae, *Hermetia illucens* in poultry by-product meal for barramundi, *Lates calcarifer* modulates histomorphology, immunity and resistance to *Vibrio harveyi*. Sci Rep. (2019) 9:16703. 10.1038/s41598-019-53018-3 31723163 PMC6853975

[22] Chaklader MR Fotedar R Howieson J Siddik MA Foysal J. The ameliorative effects of various fish protein hydrolysates in poultry by-product meal based diets on muscle quality, serum biochemistry and immunity in juvenile barramundi, *Lates calcarifer*. Fish Shellfish Immunol. (2020) 104:567–78. 10.1016/j.fsi.2020.06.014 32562869

[23] Chaklader MR Howieson J Fotedar R Siddik MA. Supplementation of *Hermetia illucens* larvae in poultry by-product meal based barramundi, *Lates calcarifer* diets improves adipocyte cell size, skin barrier functions, and immune responses. Front Nutr. (2020) 7:61358. 10.3389/fnut.2020.613158 33521040 PMC7840693

[24] Chaklader MR Howieson J Siddik MAB Foysal MJ Fotedar R. Supplementation of tuna hydrolysate and insect larvae improves fishmeal replacement efficacy of poultry by-product in *Lates calcarifer* (Bloch, 1790) juveniles. Sci Rep. (2021) 11:4997. 10.1038/s41598-021-84660-5 33654188 PMC7925588

[25] Williams KC Paterson BD Barlow CG Ford A Roberts R. Potential of meat meal to replace fish meal in extruded dry diets for barramundi, *Lates calcarifer* (Bloch). II Organoleptic characteristics and fatty acid composition *Aquacult Res*. (2003) 34:33–42. 10.1046/j.1365-2109.2003.00786.x

[26] Panicz R Zochowska-Kujawska J Sadowski J Sobczak M. Effect of feeding various levels of poultry by-product meal on the blood parameters, filet composition and structure of female tenches (*Tinca tinca*). Aquac Res. (2017) 48:5373–84. 10.1111/are.13351

[27] Siddik M Chungu P Fotedar R Howieson J. Bioprocessed poultry by-product meals on growth, gut health and fatty acid synthesis of juvenile barramundi, *Lates calcarifer* (Bloch). PLoS ONE. (2019) 14:e0215025. 10.1371/journal.pone.0215025 30964913 PMC6456252

[28] Chaklader MR Siddik MA Fotedar R. Total replacement of fishmeal with poultry by-product meal affected the growth, muscle quality, histological structure, antioxidant capacity and immune response of juvenile barramundi, *Lates calcarifer*. PLoS ONE. (2020) 15:e0242079. 10.1371/journal.pone.0242079 33180835 PMC7661056

[29] Randazzo B Zarantoniello M Gioacchini G Cardinaletti G Belloni A Giorgini E. Physiological response of rainbow trout (*Oncorhynchus mykiss*) to graded levels of *Hermetia illucens* or poultry by-product meals as single or combined substitute ingredients to dietary plant proteins. Aquaculture. (2021) 538:736550. 10.1016/j.aquaculture.2021.736550

[30] Wang Y-S Shelomi M. Review of black soldier fly (*Hermetia illucens*) as animal feed and human food. Foods. (2017) 6:91. 10.3390/foods6100091 29057841 PMC5664030

[31] Maiolo S Parisi G Biondi N Lunelli F Tibaldi E Pastres R. Fishmeal partial substitution within aquafeed formulations: life cycle assessment of four alternative protein sources. Int J Life Cycle Assess. (2020) 25:1455–71. 10.1007/s11367-020-01759-z

[32] Zarantoniello M Randazzo B Gioacchini G Truzzi C Giorgini E Riolo P. Zebrafish (*Danio rerio*) physiological and behavioural responses to insect-based diets: a multidisciplinary approach. Sci Rep. (2020) 10:1–16. 10.1038/s41598-020-67740-w 32606335 PMC7326965

[33] Nogales-Mérida S Gobbi P Józefiak D Mazurkiewicz J Dudek K Rawski M. Insect meals in fish nutrition. Rev Aquacult. (2018) 11:1080–103. 10.1111/raq.12281

[34] Bruni L Pastorelli R Viti C Gasco L Parisi G. Characterisation of the intestinal microbial communities of rainbow trout (*Oncorhynchus mykiss*) fed with *Hermetia illucens* (black soldier fly) partially defatted larva meal as partial dietary protein source. Aquaculture. (2018) 487:56–63. 10.1016/j.aquaculture.2018.01.006

[35] Osimani A Milanović V Roncolini A Riolo P Ruschioni S Isidoro N. *Hermetia illucens* in diets for zebrafish (*Danio rerio*): a study of bacterial diversity by using PCR-DGGE and metagenomic sequencing. PLoS ONE. (2019) 14:e0225956. 10.1371/journal.pone.0225956 31821372 PMC6903733

[36] Rimoldi S Gini E Iannini F Gasco L Terova G. The effects of dietary insect meal from *Hermetia illucens* prepupae on autochthonous gut microbiota of rainbow trout (*Oncorhynchus mykiss*). Animals. (2019) 9:143. 10.3390/ani9040143 30987067 PMC6523354

[37] Terova G Rimoldi S Ascione C Gini E Ceccotti C Gasco L. Rainbow trout (*Oncorhynchus mykiss*) gut microbiota is modulated by insect meal from *Hermetia illucens* prepupae in the diet. Rev Fish Biol Fish. (2019) 29:465–86. 10.1007/s11160-019-09558-y

[38] Rawski M Mazurkiewicz J Kierończyk B Józefiak D. Black soldier fly full-fat larvae meal as an alternative to fish meal and fish oil in siberian sturgeon nutrition: the effects on physical properties of the feed, animal growth performance, and feed acceptance and utilization. Animals. (2020) 10:2119. 10.3390/ani10112119 33203187 PMC7697048

[39] Chaklader MR Howieson J Fotedar R. Growth, hepatic health, mucosal barrier status and immunity of juvenile barramundi, *Lates calcarifer* fed poultry by-product meal supplemented with full-fat or defatted *Hermetia illucens* larval meal. Aquaculture. (2021) 543:737026. 10.1016/j.aquaculture.2021.73702630861487

[40] Kumar V Fawole FJ Romano N Hossain MS Labh SN Overturf K. Insect (Black soldier fly, *Hermetia illucens*) meal supplementation prevents the soybean meal-induced intestinal enteritis in rainbow trout and health benefits of using insect oil. Fish Shellfish Immunol. (2020) 109:116–24. 10.1016/j.fsi.2020.12.008 33352339

[41] Ruff N Fitzgerald R Cross T Hamre K Kerry J. The effect of dietary vitamin E and C level on market-size turbot (*Scophthalmus maximus*) fillet quality. Aquacul Nutr. (2003) 9:91–103. 10.1046/j.1365-2095.2003.00230.x

[42] Chen Y-C Nguyen J Semmens K Beamer S Jaczynski J. Effects of dietary alpha-tocopheryl acetate on lipid oxidation and alpha-tocopherol content of novel omega-3-enhanced farmed rainbow trout (*Oncorhynchus mykiss*) fillets. LWT-Food Sci Technol. (2008) 41:244–53. 10.1016/j.lwt.2007.02.016

[43] Chaklader MR Howieson J Foysal MJ Fotedar R. Transformation of fish waste protein to *Hermetia illucens* protein improves the efficacy of poultry by-products in the culture of juvenile barramundi, *Lates calcarifer*. Sci Total Environ. (2021) 796:149045. 10.1016/j.scitotenv.2021.149045 34328887

[44] AOAC. Official Methods of Analysis. 16th ed. Washington DC: Association of Official Analytical Chemists (1995).

[45] O'Fallon JV Busboom JR Nelson ML Gaskins CT. direct method for fatty acid methyl ester synthesis: application to wet meat tissues, oils, and feedstuffs. J Anim Sci. (2007) 85:1511–21. 10.2527/jas.2006-491 17296772

[46] Gedarawatte ST Ravensdale JT Johns ML Azizi A Al-Salami H Dykes GA. Effectiveness of bacterial cellulose in controlling purge accumulation and improving physicochemical, microbiological, and sensorial properties of vacuum-packaged beef. J Food Sci. (2020) 85:2153–63. 10.1111/1750-3841.15178 32572986

[47] Lawless HT Heymann H. Sensory Evaluation of Food: Principles and Practices. New York: Springer Science and Business Media (2010).

[48] Australia S. Sensory Analysis Part 1.3: Methodology-General Guidance (AS 2542.1.3:2014). (2014). Available online at: https://www.saiglobal.com (accessed May 2021).

[49] Alimentarius C. Codex Alimentarius Volume 9: Codex Guidelines for the Sensory Evaluation of Fish Shellfish in Laboratories (CAC-GL 31 – 1999). (1991). Available online at: http://www.fao.org (accessed May 2021).

[50] Kalva JJ Sims CA Puentes LA Snyder DJ Bartoshuk LM. Comparison of the hedonic general labeled magnitude scale with the hedonic 9-point scale. J Food Sci. (2014) 79:S238–45. 10.1111/1750-3841.12342 24422940

[51] Bourne MC. Texture profile analysis. Food Technol. (1978) 41:163–78.

[52] Fuentes-Amaya LF Munyard S Fernandez-Piquer J Howieson J. Sensory, microbiological and chemical changes in vacuum-packaged blue spotted emperor (*Lethrinus* sp), saddletail snapper (*Lutjanus malabaricus*), crimson snapper (*Lutjanus erythropterus*), barramundi (*Lates calcarifer*) and Atlantic salmon (*Salmo salar*) fillets stored at 4° C. Food Sci Nutr. (2016) 4:479–89. 10.1002/fsn3.309 27247777 PMC4867767

[53] Raharjo S Sofos JN Schmidt GR. Improved speed, specificity, and limit of determination of an aqueous acid extraction thiobarbituric acid-C18 method for measuring lipid peroxidation in beef. J Agric Food Chem. (1992) 40:2182–5. 10.1021/jf00023a027

[54] Ye H Zhou Y Su N Wang A Tan X Sun Z. Effects of replacing fish meal with rendered animal protein blend on growth performance, hepatic steatosis and immune status in hybrid grouper (*Epinephelus fuscoguttatus*♀ × *Epinephelus lanceolatus*♂). Aquaculture. (2019) 511:734203. 10.1016/j.aquaculture.2019.734203

[55] Dawson MR Alam MS Watanabe WO Carroll PM Seaton PJ. Evaluation of poultry by-product meal as an alternative to fish meal in the diet of juvenile Black Sea Bass reared in a recirculating aquaculture system. N Am J Aquac. (2018) 80:74–87. 10.1002/naaq.1000925855820

[56] Grigorakis K. Compositional and organoleptic quality of farmed and wild gilthead sea bream (*Sparus aurata*) and sea bass (*Dicentrarchus labrax*) and factors affecting it: a review. Aquaculture. (2007) 272:55–75. 10.1016/j.aquaculture.2007.04.062

[57] Sabbagh M Schiavone R Brizzi G Sicuro B Zilli L Vilella S. Poultry by-product meal as an alternative to fish meal in the juvenile gilthead seabream (*Sparus aurata*) diet. Aquaculture. (2019) 511:734220. 10.1016/j.aquaculture.2019.734220

[58] Caimi C Renna M Lussiana C Bonaldo A Gariglio M Meneguz M. First insights on Black Soldier Fly (*Hermetia illucens* L.) larvae meal dietary administration in Siberian sturgeon (*Acipenser baerii* Brandt) juveniles. Aquaculture. (2020) 515:734539. 10.1016/j.aquaculture.2019.734539

[59] Renna M Schiavone A Gai F Dabbou S Lussiana C Malfatto V. Evaluation of the suitability of a partially defatted black soldier fly (L.) larvae meal as ingredient for rainbow trout (Walbaum) diets. J Anim Sci Biotechnol. (2017) 8:57. 10.1186/s40104-017-0191-3 28680591 PMC5494141

[60] Stejskal V Tran HQ Prokesova M Gebauer T Giang PT Gai F. Partially defatted *Hermetia illucens* larva meal in diet of eurasian perch (*Perca fluviatilis*) juveniles. Animals. (2020) 10:1876. 10.3390/ani10101876 33066664 PMC7602402

[61] Twibell RG Gannam AL Hyde NM Holmes JS Poole JB. Effects of fish meal-and fish oil-free diets on growth responses and fatty acid composition of juvenile coho salmon (*Oncorhynchus kisutch*). Aquaculture. (2012) 360:69–77. 10.1016/j.aquaculture.2012.07.019

[62] Higgs DA Balfry SK Oakes JD Rowshandeli M Skura BJ Deacon G. Efficacy of an equal blend of canola oil and poultry fat as an alternate dietary lipid source for Atlantic salmon (*Salmo salar* L.) in sea water. I: effects on growth performance. and whole body and fillet proximate and lipid composition. Aquacult Res. (2006) 37:180–91. 10.1111/j.1365-2109.2005.01420.x

[63] Fan W Sun J Chen Y Qiu J Zhang Y Chi Y. Effects of chitosan coating on quality and shelf life of silver carp during frozen storage. Food Chem. (2009) 115:66–70. 10.1016/j.foodchem.2008.11.060

[64] Mohan C Ravishankar C Lalitha K Gopal TS. Effect of chitosan edible coating on the quality of double filleted Indian oil sardine (*Sardinella longiceps*) during chilled storage. Food Hydrocoll. (2012) 26:167–74. 10.1016/j.foodhyd.2011.05.005

[65] Farajzadeh F Motamedzadegan A Shahidi S-A Hamzeh S. The effect of chitosan-gelatin coating on the quality of shrimp (*Litopenaeus vannamei*) under refrigerated condition. Food Control. (2016) 67:163–70. 10.1016/j.foodcont.2016.02.040

[66] Baptista RC Horita CN Sant'Ana AS. Natural products with preservative properties for enhancing the microbiological safety and extending the shelf-life of seafood: a review. Food Res Int. (2020) 127:108762. 10.1016/j.foodres.2019.108762 31882098

[67] Einen O Guerin T Fjæra SO Skjervold PO. Freezing of pre-rigor fillets of Atlantic salmon. Aquaculture. (2002) 212:129–40. 10.1016/S0044-8486(01)00874-2

[68] He H-J Wu D Sun D-W. Rapid and non-destructive determination of drip loss and pH distribution in farmed Atlantic salmon (*Salmo salar*) fillets using visible and near-infrared (Vis–NIR) hyperspectral imaging. Food Chem. (2014) 156:394–401. 10.1016/j.foodchem.2014.01.118 24629986

[69] Kristoffersen S Vang B Larsen R Olsen RL. Pre-rigor filleting and drip loss from fillets of farmed Atlantic cod (*Gadus morhua* L.). Aquacul Res. (2007) 38:1721–31. 10.1111/j.1365-2109.2007.01843.x

[70] Bruni L Randazzo B Cardinaletti G Zarantoniello M Mina F Secci G. Dietary inclusion of full-fat *Hermetia illucens* prepupae meal in practical diets for rainbow trout (*Oncorhynchus mykiss*): lipid metabolism and fillet quality investigations. Aquaculture. (2020) 529:735678. 10.1016/j.aquaculture.2020.735678

[71] Iaconisi V Marono S Parisi G Gasco L Genovese L Maricchiolo G. Dietary inclusion of *Tenebrio molitor* larvae meal: effects on growth performance and final quality treats of blackspot sea bream (*Pagellus bogaraveo*). Aquaculture. (2017) 476:49–58. 10.1016/j.aquaculture.2017.04.007

[72] Siah W Ariff WM. Effect of potassium sorbate dips on refrigerated storage of modified atmosphere packed barramundi (*Lates calcarifer*) fillets. J Trop Agric Food Sci. (2003) 31:215.

[73] Wilkinson RJ Paton N Porter MJ. The effects of pre-harvest stress and harvest method on the stress response, rigor onset, muscle pH and drip loss in barramundi (*Lates calcarifer*). Aquaculture. (2008) 282:26–32. 10.1016/j.aquaculture.2008.05.032

[74] Halliwell B Gutteridge J. Free Radicals in Biology and Medicine. 3rd ed. Oxford, Oxford University Press (1999).

[75] Morrissey P Kerry J. Lipid oxidation and the shelf-life of muscle foods. Understanding and Measuring the Shelf-Life of Food. (2004). p. 357.

[76] Duun A Rustad T. Quality of superchilled vacuum packed Atlantic salmon (*Salmo salar*) fillets stored at– 1.4 and– 3.6 C. Food Chem. (2008) 106:122–31. 10.1016/j.foodchem.2007.05.051

[77] Kaale LD Eikevik TM. The influence of superchilling storage methods on the location/distribution of ice crystals during storage of Atlantic salmon (*Salmo salar*). Food Control. (2015) 52:19–26. 10.1016/j.foodcont.2014.12.022

[78] Bruni L Belghit I Lock EJ Secci G Taiti C Parisi G. Total replacement of dietary fish meal with black soldier fly (*Hermetia illucens*) larvae does not impair physical chemical or volatile composition of farmed Atlantic salmon (*Salmo salar* L). J Sci Food Agric. (2020) 100:1038–47. 10.1002/jsfa.10108 31650558

[79] Caballero M Betancor M Escrig J Montero D De Los Monteros AE Castro P. Post mortem changes produced in the muscle of sea bream (*Sparus aurata*) during ice storage. Aquaculture. (2009) 291:210–6. 10.1016/j.aquaculture.2009.03.032

[80] Ayala MD Abdel I Santaella M Martínez C Periago MJ Gil F. Muscle tissue structural changes and texture development in sea bream. *Sparus aurata* L, during post-mortem storage. LWT-Food Sci Technol. (2010) 43:465–75. 10.1016/j.lwt.2009.08.023

[81] Yu D Jiang Q Xu Y Xia W. The shelf life extension of refrigerated grass carp (*Ctenopharyngodon idellus*) fillets by chitosan coating combined with glycerol monolaurate. Int J Biol Macromol. (2017) 101:448–54. 10.1016/j.ijbiomac.2017.03.038 28283457

[82] Zarandona I López-Caballero ME Montero MP Guerrero P de la Caba K Gómez-Guillén MC. Horse mackerel (*Trachurus trachurus*) fillets biopreservation by using gallic acid and chitosan coatings. Food Control. (2021) 120:107511. 10.1016/j.foodcont.2020.107511

[83] Adachi K Kato K Wakamatsu K Ito S Ishimaru K Hirata T. The histological analysis, colorimetric evaluation, and chemical quantification of melanin content in ‘suntanned’fish. Pigment Cell Res. (2005) 18:465–8. 10.1111/j.1600-0749.2005.00272.x 16280013

[84] Finke MD. Complete nutrient composition of commercially raised invertebrates used as food for insectivores. Zoo Biol. (2002) 21:269–85. 10.1002/zoo.1003126366856

[85] Iaconisi V Bonelli A Pupino R Gai F Parisi G. Mealworm as dietary protein source for rainbow trout: body and fillet quality traits. Aquaculture. (2018) 484:197–204. 10.1016/j.aquaculture.2017.11.034

[86] Moutinho S Pedrosa R Magalhães R Oliva-Teles A Parisi G Peres H. Black soldier fly (*Hermetia illucens*) pre-pupae larvae meal in diets for European seabass (*Dicentrarchus labrax*) juveniles: effects on liver oxidative status and fillet quality traits during shelf-life. Aquaculture. (2020) 533:736080. 10.1016/j.aquaculture.2020.736080

[87] Robb D Kestin S Warriss P. Muscle activity at slaughter: I. Changes in flesh colour and gaping in rainbow trout. Aquaculture. (2000) 182:261–9. 10.1016/S0044-8486(99)00273-2

[88] Ruff N FitzGerald RD Cross TF Kerry JP. Fillet shelf-life of Atlantic halibut *Hippoglossus hippoglossus* L. fed elevated levels of α-tocopheryl acetate. Aquac Res. (2002) 33:1059–71. 10.1046/j.1365-2109.2002.00770.x

[89] Choubert G Baccaunaud M. Colour changes of fillets of rainbow trout (*Oncorhynchus mykiss* W.) fed astaxanthin or canthaxanthin during storage under controlled or modified atmosphere. LWT-Food Sci Technol. (2006) 39:1203–13. 10.1016/j.lwt.2005.06.017

[90] Guillerm-Regost C Haugen T Nortvedt R Carlehöug M Lunestad BT Kiessling A. Quality characterization of farmed Atlantic halibut during ice storage. J Food Sci. (2006) 71:S83–90. 10.1111/j.1365-2621.2006.tb08926.x

[91] Erikson U Misimi E. Atlantic salmon skin and fillet color changes effected by perimortem handling stress, rigor mortis, and ice storage. J Food Sci. (2008) 73:C50–9. 10.1111/j.1750-3841.2007.00617.x 18298716

[92] Stien LH Hirmas E Bjørnevik M Karlsen Ø Nortvedt R Rørå AMB. The effects of stress and storage temperature on the colour and texture of pre-rigor filleted farmed cod (*Gadus morhua* L.). Aquacult Res. (2005) 36:1197–206. 10.1111/j.1365-2109.2005.01339.x

[93] Ozbay G Spencer K Gill TA. Investigation of protein denaturation and pigment fading in farmed steelhead (*Onchorhychus mykiss*) fillets during frozen storage. J Food Process Preserv. (2006) 30:208–30. 10.1111/j.1745-4549.2006.00060.x

[94] Nyakeri E Ogola H Ayieko M Amimo F. An open system for farming black soldier fly larvae as a source of proteins for smallscale poultry and fish production. J Insects Food Feed. (2017) 3:51–6. 10.3920/JIFF2016.0030

[95] Luten J Martinsdottir E. QIM: a European tool for fish freshness evaluation in the fishery chain. In: Methods to Determine the Freshness of Fish in Research and Industry: Proceedings of the Final Meeting of the Concerted Action ‘Evaluation of Fish Freshness’ AIR3CT94 2283, Nantes Conference, November 12-14, 1997 (1997).

[96] Mausse ECJ Valdimarsdottir T Sveinsdottir K. Shelf Life of Red Fish Stored in Ice and Modified Atmosphere (MA) and Some Aspects on the Development of a Quality Index Method (QIM) Scheme for Red Fish Stored in MA. AD-CACS, Training Programme. Reykjavik: The United Nations University (2000).

[97] Martinsdóttir E Sveinsdottir K Luten J Schelvis-Smit R Hyldig G. Reference Manual for the Fish Sector: Sensory Evaluation of Fish Freshness. Ijmuiden: QIM Eurofish (2001).

[98] Boziaris IS. Seafood Processing: Technology, Quality and Safety. Hoboken, NJ: John Wiley and Sons (2013).

[99] Calanche J Tomas A Martinez S Jover M Alonso V Roncalés P. Relation of quality and sensory perception with changes in free amino acids of thawed seabream (*Sparus aurata*). Food Res Int. (2019) 119:126–34. 10.1016/j.foodres.2019.01.050 30884640

[100] Alexi N Hvam J Lund BW Nsubuga L de Oliveira Hansen RM Thamsborg K. Potential of novel cadaverine biosensor technology to predict shelf life of chilled yellowfin tuna (*Thunnus albacares*). Food Control. (2021) 119:107458. 10.1016/j.foodcont.2020.107458

[101] Chow C-J Yang J-I Lee P-F Ochiai Y. Effects of acid and alkaline pretreatment on the discoloration rates of dark muscle and myoglobin extract of skinned tilapia fillet during iced storage. Fisher Sci. (2009) 75:1481–8. 10.1007/s12562-009-0168-z

[102] Li XP Zhou MY Liu JF Xu YX Mi HB Yi SM. Shelf-life extension of chilled olive flounder (*Paralichthys olivaceus*) using chitosan coatings containing clove oil. J Food Process Preserv. (2017) 41:e13204. 10.1111/jfpp.13204

[103] Wang R Hu X Agyekumwaa AK Li X Xiao X Yu Y. Synergistic effect of kojic acid and tea polyphenols on bacterial inhibition and quality maintenance of refrigerated sea bass (*Lateolabrax japonicus*) fillets. LWT. (2021) 137:110452. 10.1016/j.lwt.2020.110452

[104] Gibson M Newsham P. Lipids, Oils, Fats, and Extracts. Food Science and the Culinary Arts (2018). p. 323–40.

[105] Couturier LI Michel LN Amaro T Budge SM da Costa E De Troch M. State of art and best practices for fatty acid analysis in aquatic sciences. ICES J Marine Sci. (2020) 77:2375–95. 10.1093/icesjms/fsaa121

[106] Jeon Y-J Shahidi F Kim S-K. Preparation of chitin and chitosan oligomers and their applications in physiological functional foods. Food Rev Int. (2000) 16:159–76. 10.1081/FRI-10010028625794203

[107] Feng T Du Y Li J Hu Y Kennedy JF. Enhancement of antioxidant activity of chitosan by irradiation. Carbohydr Polym. (2008) 73:126–32. 10.1016/j.carbpol.2007.11.00322222149

[108] Tsai G Su W-H Chen H-C Pan C-L. Antimicrobial activity of shrimp chitin and chitosan from different treatments. Fisher Sci. (2002) 68:170–7. 10.1046/j.1444-2906.2002.00404.x26093316

[109] Kong M Chen XG Xing K Park HJ. Antimicrobial properties of chitosan and mode of action: a state of the art review. Int J Food Microbiol. (2010) 144:51–63. 10.1016/j.ijfoodmicro.2010.09.012 20951455

[110] Kurokawa T Uji S Suzuki T. Molecular cloning of multiple chitinase genes in Japanese flounder, *Paralichthys olivaceus*. Comp Biochem Physiol B Biochem Mol Biol. (2004) 138:255–64. 10.1016/j.cbpc.2004.03.015 15253874

[111] Fines B Holt G. Chitinase and apparent digestibility of chitin in the digestive tract of juvenile cobia, *Rachycentron canadum*. Aquaculture. (2010) 303:34–9. 10.1016/j.aquaculture.2010.03.010

[112] Varlik C. Su urunlerinde kalite kontrol ilke ve yontemleri. Gida Teknolojisi Dernegi. (1993) 17:16–7.

[113] Öz M. Effects of garlic (*Allium sativum*) supplemented fish diet on sensory, chemical and microbiological properties of rainbow trout during storage at– 18 C. LWT. (2018) 92:155–60. 10.1016/j.lwt.2018.02.030

